# Cell-permeable peptide nucleic acid antisense oligonucleotide platform targeting human betacoronaviruses

**DOI:** 10.3389/fmicb.2023.1258091

**Published:** 2023-09-29

**Authors:** Soree Park, Seong Ho Kim, Mehrangiz Dezhbord, Eun-Hwi Lee, Yeasel Jeon, Daram Jung, Se Hun Gu, Chiho Yu, Seung Ho Lee, Sung Chun Kim, Kyun-Hwan Kim

**Affiliations:** ^1^Department of Precision Medicine, Sungkyunkwan University School of Medicine, Suwon, Republic of Korea; ^2^OliPass Inc., Yongin, Gyeonggi, Republic of Korea; ^3^Chem-BioTechnology Center, Advanced Defense Science & Technology Research Institute, Agency for Defense Development, Daejeon, Republic of Korea

**Keywords:** SARS-CoV-2, antisense oligonucleotide, human betacoronavirus, modified PNA, cell-permeable peptide nucleic acid

## Abstract

**Introduction:**

Antisense oligonucleotides (ASOs) with therapeutic potential have recently been reported to target the SARS-CoV-2 genome. Peptide nucleic acids (PNAs)-based ASOs have been regarded as promising drug candidates, but intracellular delivery has been a significant obstacle. Here, we present novel modified PNAs, termed OPNAs, with excellent cell permeability that disrupt the RNA genome of SARS-CoV-2 and HCoV-OC43 by introducing cationic lipid moiety onto the nucleobase of PNA oligomer backbone.

**Methods:**

HCT-8 cells and Caco-2 cells were treated with 1 μM antisense OPNAs at the time of viral challenge and the Viral RNA levels were measured by RT-qPCR three days post infection.

**Results:**

NSP 14 targeting OPNA 5 and 11, reduced the viral titer to a half and OPNA 530, 531 and 533 lowered viral gene expression levels to less than 50% of control by targeting the 5’ UTR region. Several modifications (oligo size and position, etc.) were introduced to enhance the efficacy of selected OPNAs. Improved OPNAs exhibited a dose-dependent reduction in viral replication and nucleoprotein (NP) protein. When a mixture of oligomers was applied to infected cells, viral titer and NP levels decreased by more than eightfold.

**Discussion:**

In this study, we have developed a modified PNA ASO platform with exceptional chemical stability, high binding affinity, and cellular permeability. These findings indicate that OPNAs are a promising platform for the development of antivirals to combat future pandemic viral infections that do not require a carrier.

## 1. Introduction

The coronavirus family includes a number of well-known viruses, including the severe acute respiratory syndrome coronavirus 2 (SARS-CoV-2), responsible for the recent COVID-19 pandemic, as well as other viruses that caused several pandemics before high mortality rate, such as SARS-CoV and Middle East respiratory syndrome coronavirus (MERS-CoV) (Wu and McGoogan, 2020; Zhu et al., 2020; Pustake et al., 2022; Vora et al., 2022). These viruses with intense pathogenicity are enveloped viruses consisting of relatively large positive-sense RNA genomes that are responsible for encoding a number of structural proteins and many more non-structural proteins (NSPs) (Dolliver et al., 2022).

Aside from the vaccines readily used to control the spread of the virus, other methods are required to combine with preventive therapy to speed up the treatment process and reach complete cure (Robson et al., 2020).

Antisense oligonucleotides (ASOs) are a type of oligonucleotide-based drugs that work by binding to specific RNA molecules and regulating the target gene expression. They are synthetic, single-stranded RNA or DNA molecules that are designed to be complementary to the target RNA sequence.

ASOs have been studied for the treatment of a wide range of diseases, including cancer, genetic disorders, and viral infections (Delcroix and Riley, 2010; Ahn et al., 2011; Tarn et al., 2021; Quemener et al., 2022). One example of an ASO drug that has been approved for clinical administration is nusinersen (Spinraza) (Finkel et al., 2016), which is used to treat spinal muscular atrophy, a rare genetic disorder that affects muscle strength and movement. Another example is inotersen (Tegsedi) (Benson et al., 2018), which is used to treat hereditary transthyretin-mediated amyloidosis, a genetic disorder that can cause damage to the nerves, heart, and other organs.

ASOs have also been studied for the treatment of COVID-19. A number of studies have been conducted worldwide to investigate the practical applicability of ASOs in blocking the replication of the SARS-CoV-2 virus by targeting specific viral RNA sequences. ASOs could potentially be used in combination with other drugs to treat COVID-19 or prevent the transmission of the virus (Hegde et al., 2021; Lulla et al., 2021; Pfafenrot et al., 2021; Dhorne-Pollet et al., 2022; Quemener and Galibert, 2022; Vora et al., 2022; Zhu et al., 2022).

In an attempt to improve the properties of ASOs such as their affinity to DNA and RNA, the originally developed oligomers have been modified with different chemical groups, and as a result, their feasibility has dramatically risen over the past decades. Based on these alternative DNA modifications, some previously designed ASOs have been developed for therapeutic purposes and have entered Phase III clinical trial (Rinaldi and Wood, 2018). Two main representatives of this optimized type of oligonucleotides are locked nucleic acids (LNAs), and peptide nucleic acids (PNAs).

PNAs constitute a remarkable class of nucleic acid mimics, with important physiochemical properties that have been exploited to develop a wide range of powerful biomolecular tools (Pellestor and Paulasova, 2004).

PNA which was first developed by Dr. Nielsen has a similar structure to DNA and RNA but with a peptide-like backbone instead of a sugar-phosphate backbone (Nielsen et al, 1991). PNAs have a high binding affinity for complementary DNA and RNA sequences, and it is resistant to degradation by nucleases (Pellestor and Paulasova, 2004). Moreover, PNAs have improved hybrid duplex stability compared with traditional RNA or DNA, which makes it more resistant to degradation *in vitro* and *in vivo*. However, PNA also has some limitations such as poor cell permeability and physicochemical properties, so they have been used primarily for diagnostic purposes and cannot yet be druggable.

In this study, we developed modified PNAs with excellent cell permeability (termed OPNA), which was derived from PNA by introducing cationic lipid moiety into nucleobase.

OPNA not only maintains binding affinity and stability but also overcomes the limitations of poor cell delivery of PNA by eliminating the need for carrier or vehicle molecules.

Our ultimate goal was to develop a cell-permeable PNA ASO platform that targets RNA viruses, eliminating the need for a delivery vehicle. As a model system, we interrupted HCoV-OC43 and SARS-CoV-2 genome by targeting its conserved regions with optimized PNA oligomers (OPNA) to reduce virus replication.

Therefore, the antisense and sense oligonucleotides were designed and produced to specifically target the conserved sequences including the 5’ UTR region of SARS-CoV-2 and HCoV-OC43. The comprehensive screening was conducted to identify the best oligomer candidates capable of practically inhibiting the virus titer in our infection system. Our experimental results reveal potent OPNAs exhibiting strong antiviral impact, particularly when treated in a combination and as a mixture of oligos targeting different generic regions of the virus. Furthermore, we show successful binding of designed OPNAs to NSP14 of SARS-CoV-2 causing a significant reduction in its expression levels. Most importantly, in our study, by focusing on recent efforts to improve oligonucleotide drug delivery, we established a novel modified PNA ASO platform that is efficient in targeting coronaviruses, which could be used as an additional therapeutic strategy to overcome the complications of the disease in the near future.

## 2. Materials and methods

### 2.1. Synthesis of modified peptide nucleic acid oligomers

Antisense and sense oligonucleotides targeting the conserved sequences of HCoV-OC43 (NC_006213.1) and SARS-CoV-2 (MT039890.1) were designed (Supplementary Table S1 and Supplementary Figure S1). We designed different OPNA oligomers targeting ORF1a, ORF1b, and the 5′ untranslated region (UTR) sequence.

OPNA oligomers were synthesized by solid-phase peptide synthesis (SPPS) based on Fmoc chemistry according to the method described in our previous patent (Chung et al., 2014, 2017), with minor modifications. The H-Rink Amide-ChemMatrix, which was employed as a solid support, was purchased from PCAS BioMatrix Inc. (Quebec, Canada). Fmoc-PNA monomers with a modified nucleobase were synthesized as described previously (Chung et al., 2014, 2017). Modified nucleobase and natural Fmoc-PNA monomers were used to synthesize the PNA derivatives of this study. PNA oligomers were purified by C18-reverse phase HPLC (water/acetonitrile or water/methanol with 0.1% TFA) and characterized by mass spectrometry including ESI/TOF/MS.

The concentration of 1 μM was decided for the initial screening of the designed OPNA. Tables, including all the details related to the OPNAs of this study, are presented in Supplementary Section.

### 2.2. Cell culture, transfection, and OPNA treatment

HCT-8 cells, Caco-2 cells, and Vero E6 cells were obtained from the American Type Culture Collection (ATCC, Manassas, VA, United States). HCT-8 cells were maintained in RPMI medium supplemented with 10% FBS and penicillin/streptomycin at 37°C for 3 days in a 5% CO2 incubator. Caco-2 cells and Vero E6 cells were maintained in Dulbecco’s modified Eagle’s medium (DMEM) with 10% FBS and penicillin/streptomycin at 37°C for 3 days in a 5% CO2 incubator. OPNA, PF07321331 (Merck, NJ, United States), EIDD-1931(Merck, NJ, United States), chloroquine (Merck, NJ, United States), and kurarinone (Merck, NJ, United States) were treated to the cells at the indicated concentrations. The plasmid (myc-NSP14) was transfected with Lipofectamine 2000 transfection reagents (Invitrogen, MA, United States). The siRNA (N14-R7) (Gu et al., 2020) transfection was performed with Lipofectamine RNAiMAX transfection reagents (Invitrogen, MA, United States), according to the manufacturer’s instructions. The siRNA (5′- AAAGACAUCAGCAUACUCCdTdT-3′) was designed to target the RdRp sequence at 5’-GGAGUAUGCUGAUGUCUUU-3′.

### 2.3. Virus preparation and infection

HCoV-OC43 was gathered from the supernatant of HCT-8 cells at 4 to 5 days post-infection. The prepared HCoV-OC43 was used to infect HCT-8 or Caco-2 cells with serum-free medium for 30 min. After 30 min, cells were washed using serum-free fresh medium and maintained in media supplemented with 2% FBS and penicillin/streptomycin. SARS-CoV-2 viruses were propagated in Vero E6 cells and maintained in DMEM supplemented with 2% heat-inactivated fetal bovine serum (FBS; GIBCO, United States). All experimental procedures related to SARS-CoV-2 were performed in a Biosafety Level 3 (BSL-3) facility with the approval of the Agency for Defense Development.

### 2.4. Viral genome extraction and quantitative real-time PCR

The viral RNA from the cell culture supernatants was isolated using the viral RNA purification QIAamp Kit (Qiagen, Hilden, Germany). The DNA from transfected cells was isolated using TRI Reagent (Merck, NJ, United States), according to the manufacturer’s protocol. Quantitative reverse transcription PCR (RT-qPCR) was performed using the One Step SYBR^®^ PrimeScriptTM RT-PCR Kit (Takara Bio Inc., Shiga, Japan), according to the manufacturer’s protocol using primer pairs to amplify the HCoV-OC43 nucleoprotein (NP) gene (forward, 5′ -AGCAACCAGGCTGATGTCAATACC-3′, and reverse, 5′ -AGCAGACCTTCCTGAGCCTTCAAT-3′). For amplifying RdRp of SARS-CoV-2, the following primers and probes were used: forward primer, 5’-GTGARATGGTCATGRGTGGCGG-3′; reverse primer, 5’-CARATGTTAAASACACTATTAGCATA-3′; probe, FAM-CAGGTGGAACCTCATCAGGAGATGC-BBQ. For amplifying NSP14 of SARS-CoV-2, the following primers were used: forward primer, 5’-TGGGGCTTTACGGGTAACCT-3′; reverse primer, 5’-AACACGCTTAACAAAGCACTC-3′.

### 2.5. Fifty-percent tissue culture infective dose assay

HCoV-OC43 titers were analyzed using TCID_50_ assay in HCT-8 cells. In brief, 2,000 cells were plated in each well of 96-well plates. Medium from HcoV-OC43-infected cells was used for half-log serial dilution with RPMI and added to the cells. The plates were observed for cytopathic effect (CPE) at 4 or 5 days after infection. The TCID_50_ results were measured using Viral ToxGlo™ (Promega, United States), according to the manuals. The TCID_50_ results were calculated using the Spearman and Karber methods.

### 2.6. Inhibitory concentration analysis

The half maximal inhibitory concentration (IC_50_) was analyzed by two different methods as follows: (1) the viral titer in cell supernatants by RT-QPCR and (2) the TCID_50_ values which were measured using Viral ToxGlo™ (Promega, United States). IC_50_ values were obtained by non-linear regression analysis using GraphPad Prism^®^ software V.6.05 for Windows (GraphPad Software Inc., San Diego, CA, United States).

### 2.7. Western blot analysis

SDS-PAGE and Western blot analysis were performed as described previously (Park et al., 2020). In brief, cells were lysed in a lysis buffer (M-per and a protease inhibitor cocktail) and centrifuged at 3 days after infection or 2 days after transfection. Cell lysates were separated in 12% SDS-PAGE gels and subjected to Western blot analysis. The membranes were incubated with specific antibodies, such as antiviral-NP (Merck, NJ, United States), anti-Myc (Cell Signaling Technology, MA, United States), anti-GFP (Clontech, CA, United States), and anti-β-actin (Merck, NJ, United States).

Chemiluminescent detection was performed with the ECL Detection Reagent (AbClon, Seoul, Republic of Korea) and visualized with a Bio-Imaging analyzer (GE Healthcare, Buckinghamshire, United Kingdom).

### 2.8. Immunofluorescence analysis

HCT-8 cells were seeded in a glass bottom 8-well cell culture slide (SPL, South Korea) and incubated overnight. Next, the FAM-labeled OPNA was treated for 30 min before washing with serum-free RPMI. The cell nuclei were stained with DAPI for 20 min. The slide was observed with a confocal microscope (Leica, Germany), and data analysis was performed using Leica Application Suite X.

### 2.9. Cell viability analysis

HCT-8 cells were seeded in a 96-well clear-bottom white cell culture plate. Next day, OPNAs were treated with RPMI containing 10% FBS and 1% penicillin/streptomycin in a dose-dependent manner. The cell viability was measured using a CellTiter-Glo^®^ Luminescent Cell Viability Assay Kit on the second day following treatment. An equal volume of reagent was added; plates were shaken for 2 min on an orbital shaker and incubated for 10 min at room temperature. Next, the luminescence signal was detected using the GloMax^®^ 96 Microplate Luminometer. The values were obtained by non-linear regression analysis using GraphPad Prism^®^ Software V.6.05 for Windows (GraphPad Software Inc., San Diego, CA, United States).

### 2.10. Statistical analyses

Data were presented as mean values, and error bars were presented with standard deviation (SD). Data analysis was performed using GraphPad Prism 6. Data were analyzed using two-tailed unpaired Student’s *t*-test. All statistical analyses were two-sided, and a *p*-value of <0.05 was considered statistically significant.

## 3. Results

### 3.1. Chemical structure and cell permeability of OPNA

**Figure 1:**
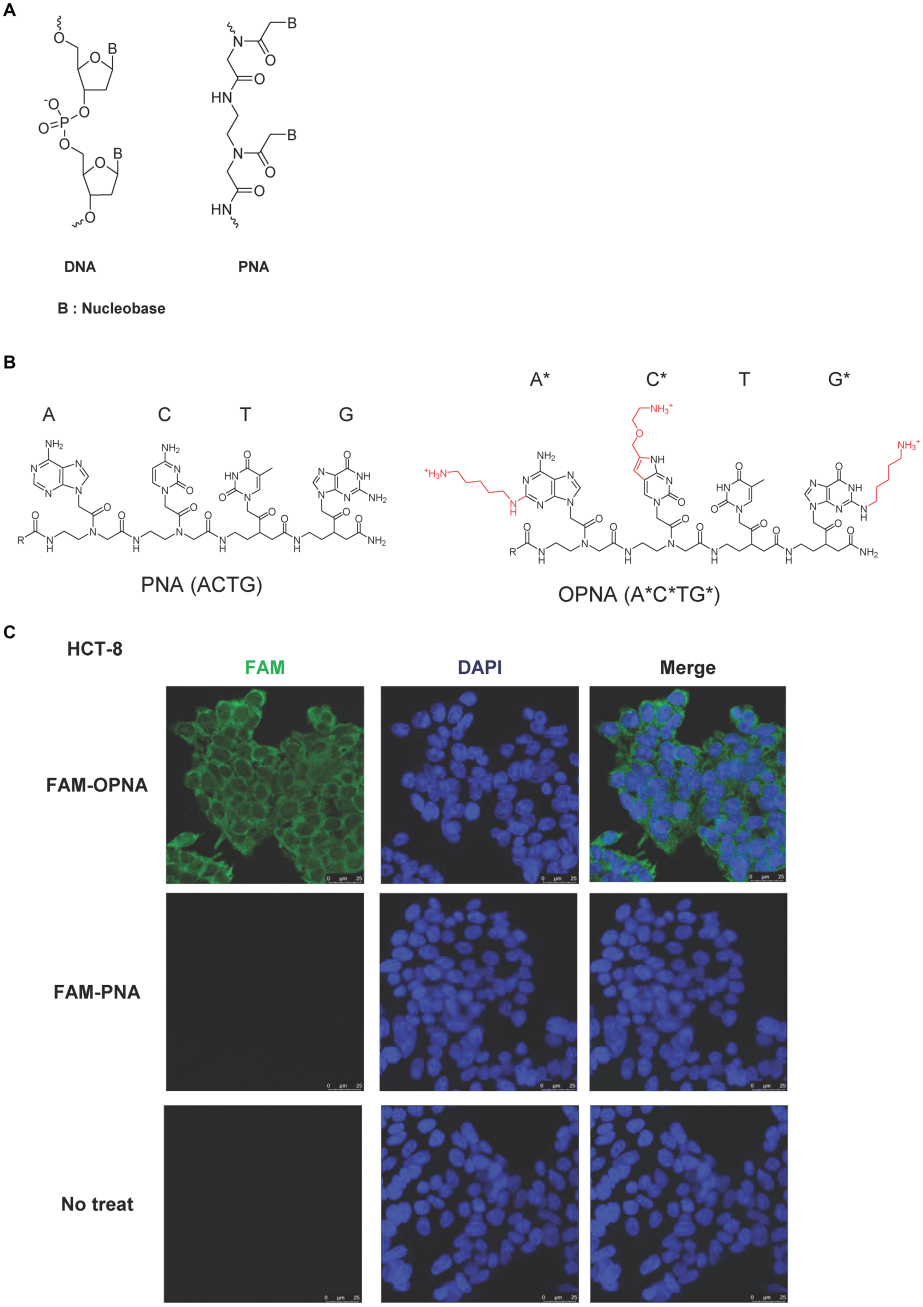
Chemical structure of ASOs and permeability of OPNAs in HCT-8 cells. **(A)** Schematic representation of DNA and PNA. **(B)** Chemical structure of PNA and modified PNA (OPNA). Three lipid modifications, located on the nucleobase of adenine (A), cytosine (C), and guanine (G), were marked in red with asterisks or numbers. **(C)** Cell permeability of FAM-OPNA in HCT-8 cells. HCT-8 cells were seeded in an 8-well cell culture slide and incubated overnight. FAM-OPNA was treated at a concentration of 1 μM for 30 min, and nuclei were stained with DAPI for 20 min. Images were obtained by a confocal laser microscope.

The chemical structure of well-studied DNA archetypes, LNA, and PNA is presented in Figure 1A. We have developed novel PNA-based ASOs, so-called OPNAs, designed to sterically block the access of proteins or nucleic acids to the RNA by introducing cationic lipid moiety into the nucleobase of PNA oligomer backbone (Figure 1B). Three lipid modifications that are located on the nucleobase of adenine (A), cytosine (C), and guanine (G) are marked in red with asterisks. In this study, to assess the cell permeability of a designed 14-mer OPNA incorporating 6 modified nucleobases, HCT-8 cells were employed. These cells are commonly used for HCoV-OC43 infection evaluation. Treatment involved the administration of FAM-labeled OPNA or PNA to the cells (Figure 1C). Surprisingly, fluorescence was fully detected only in cells treated with OPNA oligomers, and all cells were detected, resulting in 100% cell permeability. Conventional PNA, however, barely penetrated the cells.

### 3.2. Antisense OPNA oligomers inhibit the replication of HCoV-OC43

**Figure 2:**
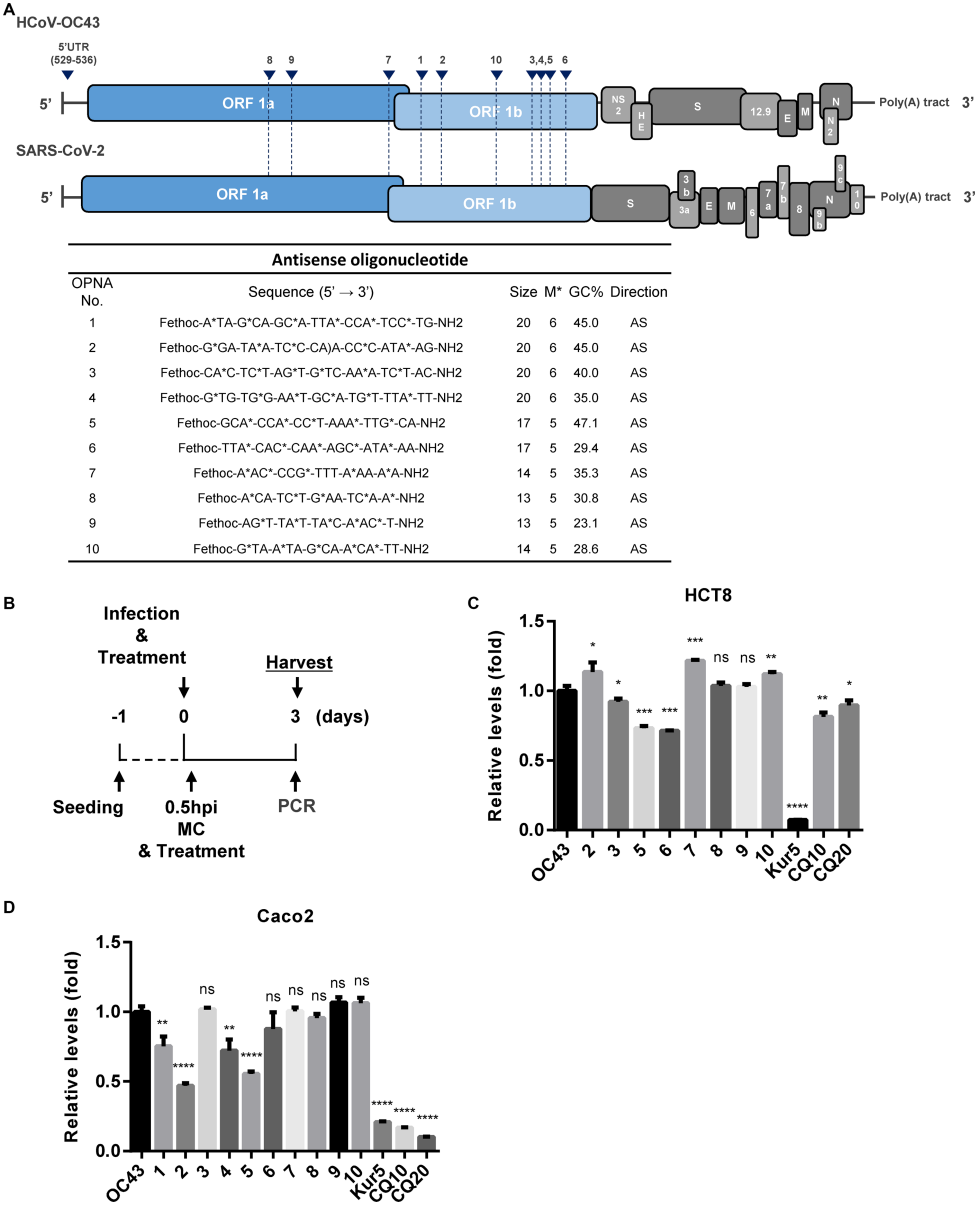

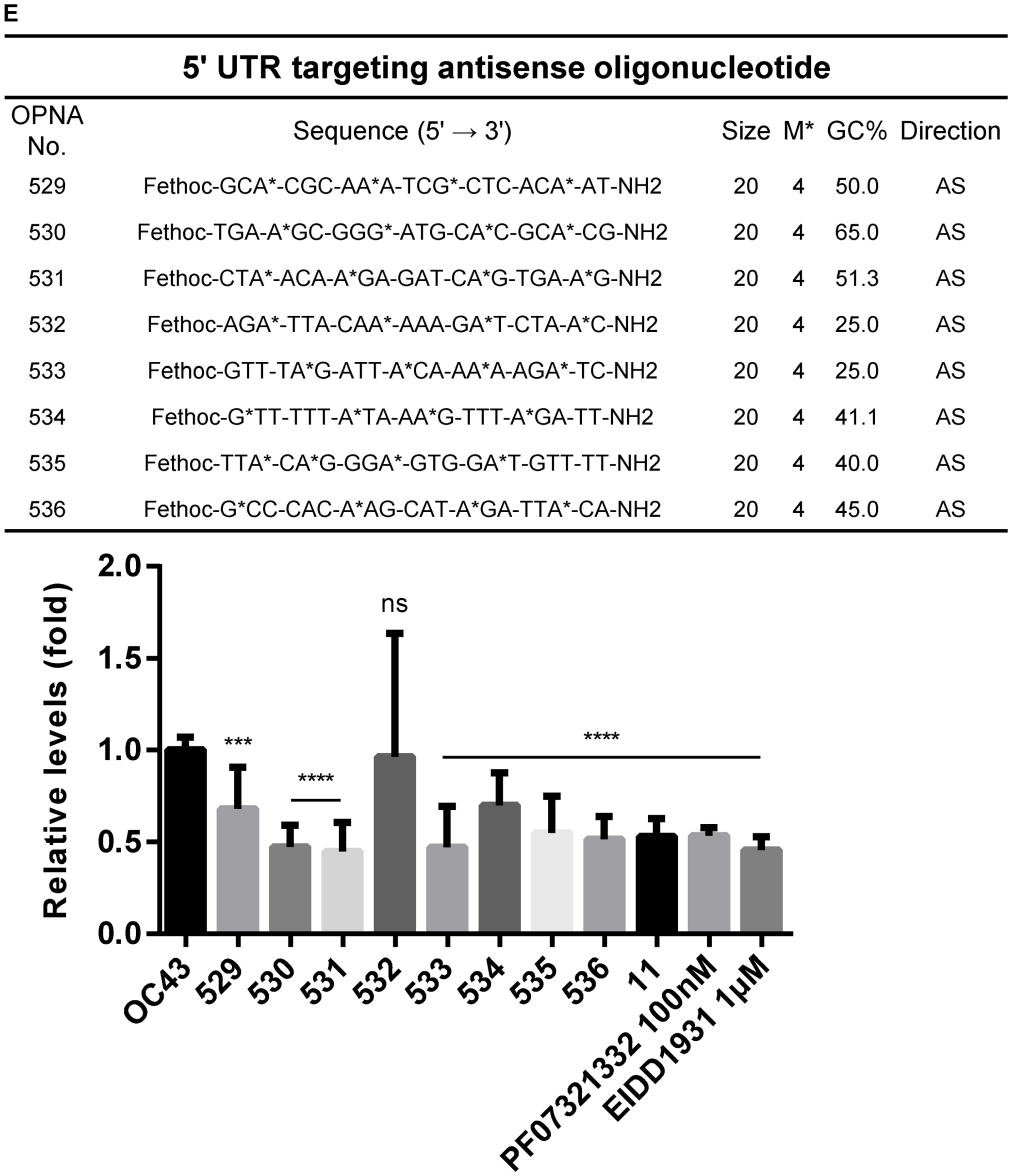
Design of antisense OPNA oligomers and inhibition of HCoV-OC43. **(A)** Antisense OPNA oligomers design for conserved regions of HCoV-OC43 and SARS-CoV-2. The arrows indicate the binding site of designed OPNAs. AS, antisense. **(B)** Scheme of virus inhibition assay and OPNA treatment. MC, media change. **(C)** Virus inhibitory effect test of OPNA in HCT-8 cells. In a 6-well plate, HCT-8 cells were treated with OPNA (1 μM) and were infected with 1 × 10^3^ TCID_50_ of HCoV-OC43 virus. Kurarinone was treated at 5 μM and chloroquine was treated at 10 or 20 μM. Cell culture medium was collected at 3 dpi. For OPNA screening, viral RNA levels were analyzed by RT-qPCR. **(D)** OPNA screening in Caco-2 cells. Caco-2 cells were treated with OPNAs (1 μM) and then infected with 1 × 104 TCID_50_ of HCoV-OC43 virus on a 6-well plate. Viral RNA levels in the culture medium were analyzed by RT-qPCR at 3 dpi. **(E)** Design of 5’UTR targeting antisense OPNA oligomers and confirmation of OC43 virus inhibitory effect. In a 6-well plate, HCT-8 cells were infected with 5 × 10^2^ TCID_50_ of HCoV-OC43 virus. Cells were treated with OPNA or EIDD1931 at 1 μM and PF07321332 at 100 nM. For C to E, data were presented as means ± SEM of three independent experiments, and Student’s *t*-test was used to determine significance. OC43 (control) versus OPNA-treated group in each experiment, symbols represent mean ± SD. ** *p* < 0.01, *** *p* < 0.001, **** *p* < 0.0001, *p* > 0.05; ns, not significant.

Initially, the genomic sequences of human coronaviruses, HCoV-OC43 and SARS-CoV-2, were aligned, and the generic conserved sequences between them were identified. Next, multiple OPNA ASOs targeting these particular regions were synthesized and evaluated for their antiviral activity against infection with the human coronavirus, HCoV-OC43. The oligomer structure of these OPNA ASOs is presented in Figure 2A.

To find out that the manufactured OPNAs can inhibit viral replication *in vitro*, experiments were conducted in HCT-8 cells and Caco-2 cells, according to the scheme mentioned in Figure 2B. We combined co-treatment and post-treatment experiments to obtain optimal treatment time course. Cell culture medium was collected at 3 days post-infection (dpi) for the quantification of HCoV-OC43 replication level as described in Materials and Methods. RT-qPCR data showed that the OPNA 5, which targets the NSP14, reduced the viral titer in both cells (Figures 2C,D). Kurarinone (Min et al., 2020) which reportedly inhibited HCoV-OC43 infection and chloroquine (Singh et al., 2021) was used as a positive control. In general, antiviral effects were more pronounced in Caco-2 cells than in HCT-8 cells. Additionally, since the 5’ UTR region plays an important role in the life cycle of the virus, we designed and synthesized the OPNAs against this region to investigate their antiviral effect. *In vitro* screening of ASO candidates targeting the 5’ UTR region exhibited similar antiviral impact. Especially, OPNA 530, 531, and 533 lowered viral gene expression to half which is comparable with the inhibitory effect of the previously described EIDD1931 (parent nucleoside of molnupiravir) (Rosenke et al., 2021) and PF07321332 (viral 3CL protease inhibitor developed by Pfizer) (Owen et al., 2021) (Figure 2E). The above data confirmed the antiviral effect of OPNA ASOs against coronavirus in our *in vitro* cell system.

### 3.3. Sense OPNA oligomers interrupted the replication of HCoV-OC43

**Figure 3:**
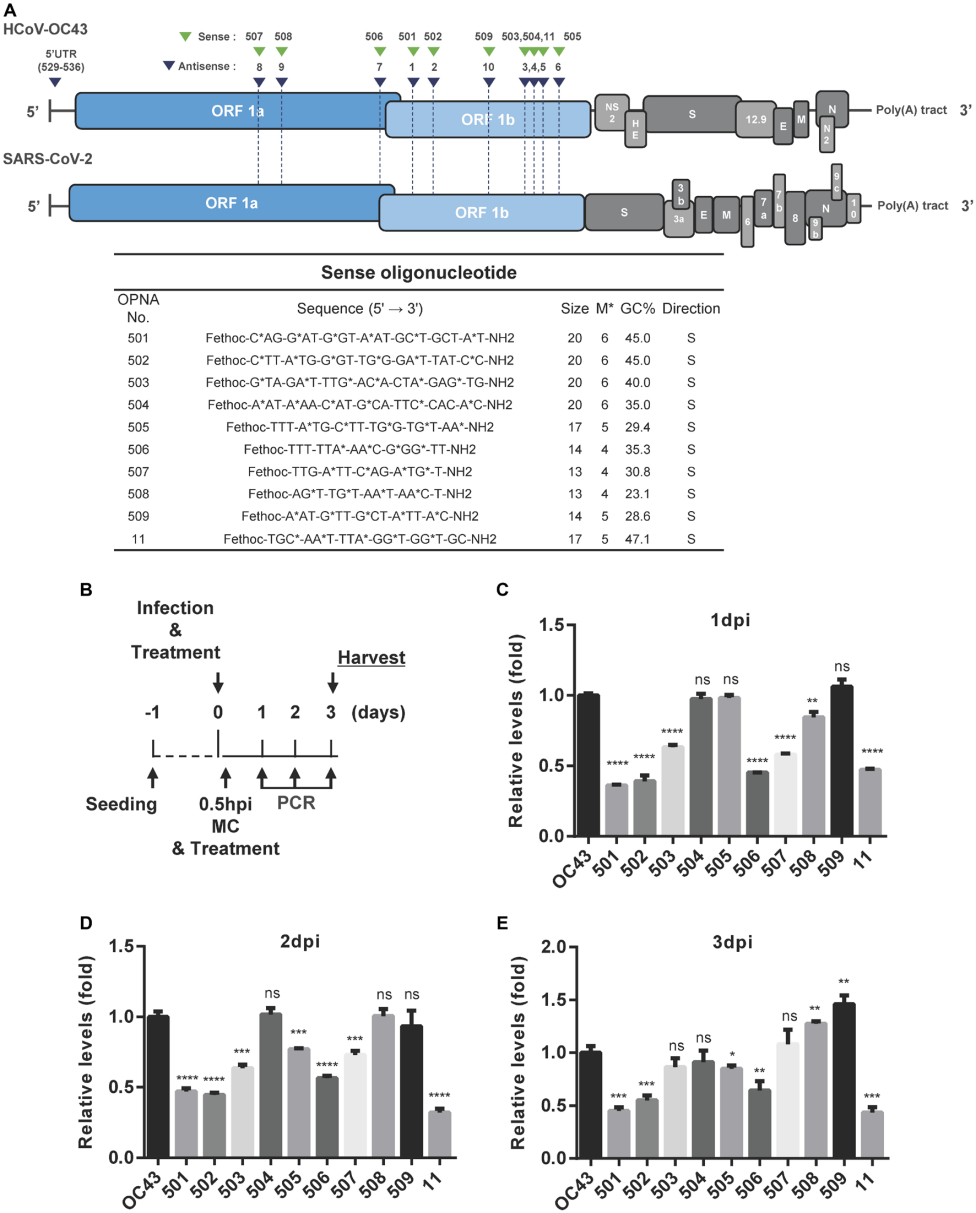
Design of sense OPNA oligomers and antiviral effect of OPNAs. **(A)** Sense OPNA oligomers were designed to target conserved regions of HCoV-OC43 and SARS-CoV-2. The green arrows indicate the binding site of sense OPNAs, and the blue arrows indicate the binding site of antisense OPNAs. S, sense. **(B)** Scheme of virus inhibition assay by OPNA treatment. MC, media change; Hpi, hour post-infection. **(C–E)** Antiviral effect test of OPNAs in HCT-8 cells for 3 days after infection. In a 6-well plate, HCT-8 cells were infected with the HCoV-OC43 virus at 5 × 10^2^ TCID_50_, and OPNA was treated at 1 μM. Cell culture medium was collected at 1, 2, and 3 dpi. Viral RNA levels were analyzed by RT-qPCR for OPNA screening. For **(C–E)**, data were presented as means ± SEM of three independent experiments, and Student’s *t*-test was used to determine the significance. OC43 (control) versus OPNA-treated group in each experiment, symbols represent mean ± SD. ** *p* < 0.01, *** *p* < 0.001, **** *p* < 0.0001, *p* > 0.05; ns, not significant.

A similar strategy was used to design sense OPNA oligomers targeting the HCoV-OC43 genome. Sense OPNA oligomers which are complementary to the ASOs were developed, as shown in Figure 3A, and their viral inhibitory effect was evaluated according to the scheme presented in Figure 3B. The antiviral activity of sense OPNA oligomers was monitored daily after OPNA treatment for 3 days post-infection (Figures 3C–E). According to the RT-qPCR results, sense OPNA oligomers (OPNA 501, 502, and 506) comparably diminished virus replication in HCT-8 cells. Interestingly, sense OPNA 11, which is complementary to OPNA 5, was found to be particularly effective in suppressing the virus in the early stages of infection, reducing viral titer to less than 50% at dpi. These results suggested that both sense and antisense OPNA oligomers are capable of inhibiting HCoV-OC43 in HCT-8 cells.

### 3.4. Inhibition of HCoV-OC43 by OPNA 11- and OPNA 5-based modifications

**Figure 4:**
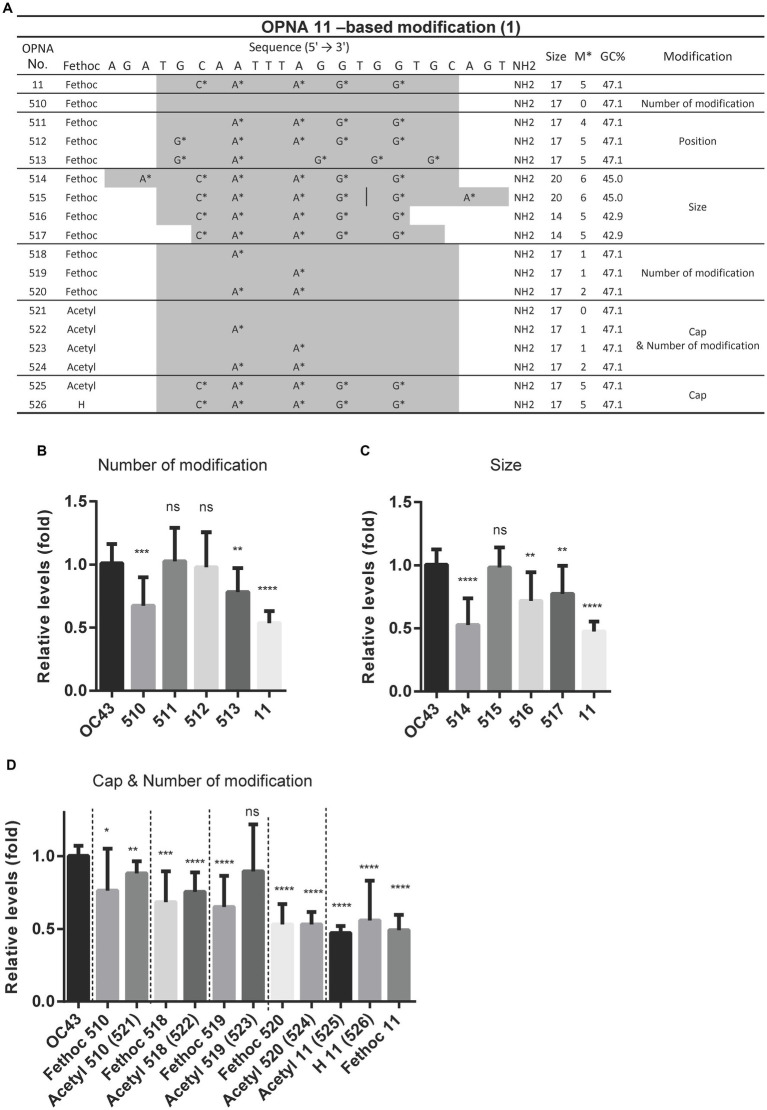

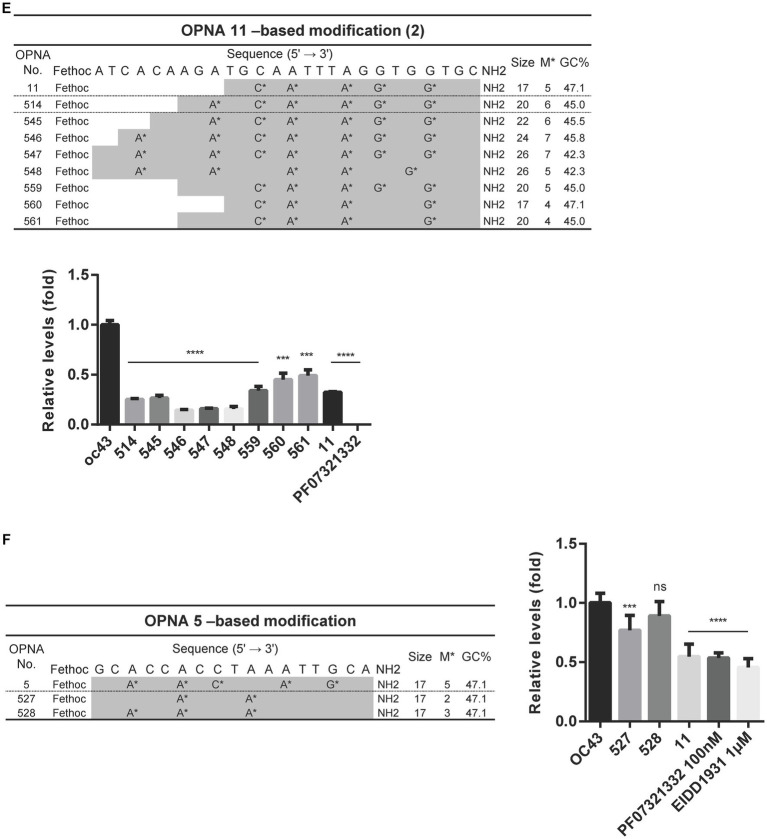
Attenuating HCoV-OC43 by OPNA 11- and OPNA 5-based modifications. **(A)** Design of OPNA11-based modification (1). The OPNA sequences are marked in gray. **(B–D)** Inhibition of viral replication by OPNA 11-based modification by RT-qPCR. **(E)** The target sequence and virus inhibitory effect of OPNA 11-based modification (2). The modified OPNA sequences are marked in gray. **(F)** Virus inhibitory effect test of OPNA 5-based modified OPNAs. The OPNA sequences are marked in gray. For **(B–F)**, HCT-8 cells were infected with HCoV-OC43 virus at 500 TCID_50_, and OPNAs were treated at 1 μM in a 6-well plate. Cell supernatant was collected at 3 dpi for titration. Data were presented as means ± SEM of three independent experiments, and Student’s *t*-test was used to determine the significance. OC43 (control) versus OPNA-treated group in each experiment, symbols represent mean ± SD. ** *p* < 0.01, *** *p* < 0.001, **** *p* < 0.0001, *p* > 0.05; ns, not significant.

As the sense OPNA oligomer 11 exhibited the highest antiviral impact, it was selected for further modifications to enhance its efficiency. A variety of modifications were explored including oligomer size, the number and position of each modification, and lastly attaching a cap to the original OPNA 11 structure (Figure 4A). In the case of the number of modifications, the most effective OPNA 11 was the one containing five modifications. Nevertheless, different numbers and types of modifications or changes in oligomer size did not seem to positively enhance the effect of OPNA 11. Elongating the OPNA from 17 mer (OPNA 11) to 20 mer (OPNA 514 and 515) resulted in a similar reduction level in OC43 expression level (Figures 4B,C).

In addition to the fethoc cap that was initially introduced in OPNA 11, other options including acetyl or H groups were substituted, and relative antiviral activity was measured. Apparently, the OPNAs with a small number of modifications and the fethoc cap better enhanced the antiviral feature of OPNA than acetyl or H (OPNA 525 vs. 526 vs. 11). However, when the number of modifications was 2 (OPNA 520 vs. 524) or 5 (OPNA 11 vs. 525 vs. 526), the different types of cap had a consistent effect without much difference (Figure 4D).

Increasing the number of modifications and extending the oligomer at the 5′ end showed room for improvement in the performance of OPNA 11 (OPNA 514 vs. 11). Therefore, we further modified the OPNA 11 in this order and evaluated its antiviral ability in HCoV-OC43-infected HCT-8 cells (Figure 4E). OPNA 456 reduced viral replication most significantly (approximately 80%). In addition, OPNA 5, a complementary sequence to OPNA 11, had relatively good antiviral activity among the antisense OPNAs (Figure 2), so we modified it to compare its effectiveness with OPNA 11. However, as shown in the activity data in Figure 4F, the OPNA 5-based modifications (OPNAs 527 and 528) were unable to compete with OPNA 11 (Figure 4F). Overall, our data suggest that the antiviral effect can be modulated by controlling the length, number of modifications, position, and cap of the OPNA. Based on the optimization results, OPNAs 11 and 546 have been modified to exert maximum antiviral effect on our system.

### 3.5. Optimized OPNAs that selectively target the 5’UTR of HCoV-OC43 inhibit the virus

**Figure 5:**
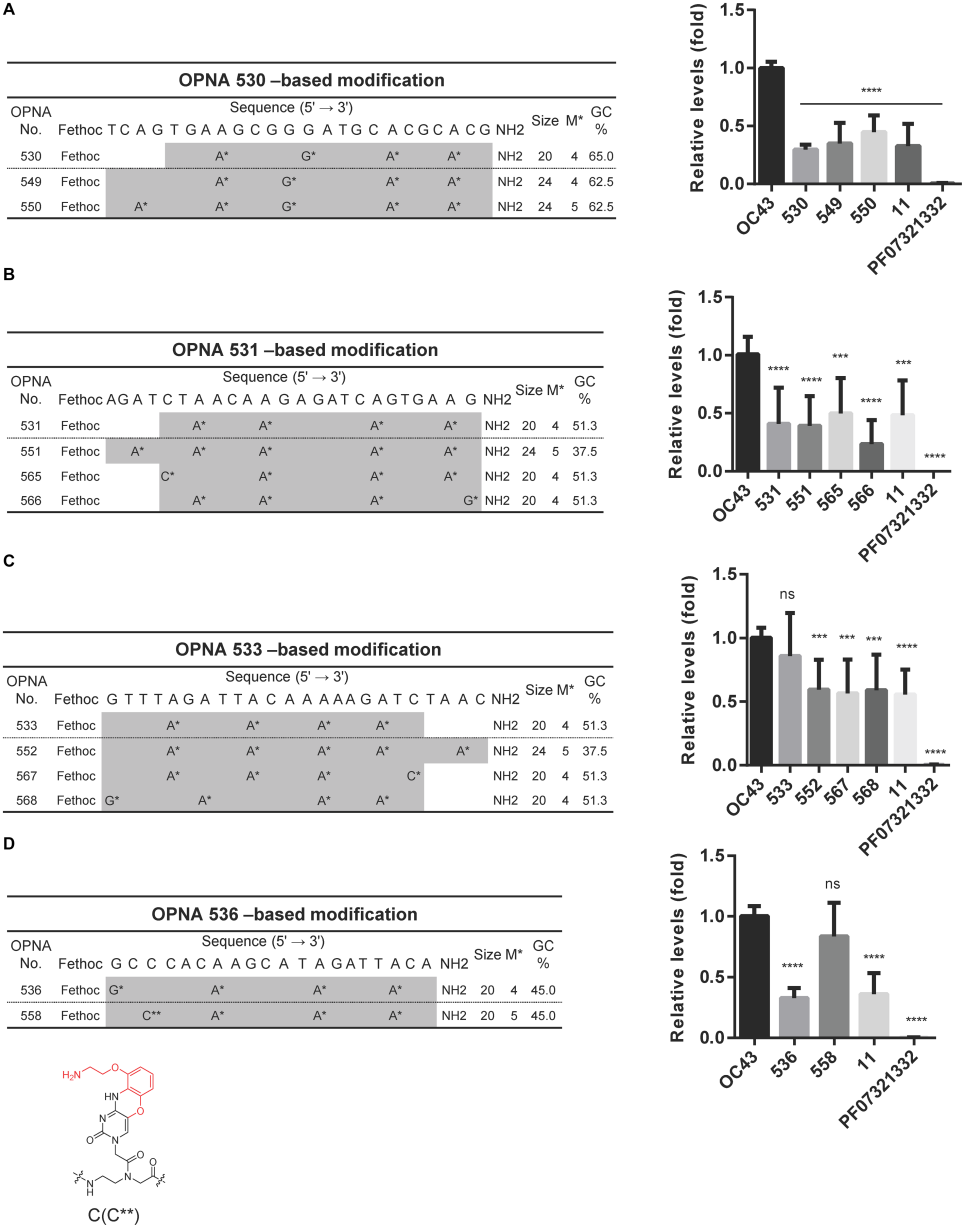
Reduction of HCoV-OC43 replication by modified OPNAs targeting 5’UTR. **(A–D)** The sequences of OPNA 530, 531, 533, and 536-based modifications located in the 5’ UTR region and their inhibitory effect by RT-qPCR. The OPNA sequences are marked in gray. In a 6-well plate, HCT-8 cells were infected with 500 TCID_50_ of HCoV-OC43 virus, and OPNA was treated at 1 μM. PF07321332 was treated at 1 μM. Cell supernatant was collected at 3 dpi for the experiment. Data were presented as means ± SEM of three independent experiments, and Student’s *t*-test was used to determine the significance. OC43 (control) versus OPNA-treated group in each experiment, symbols represent mean ± SD. ** *p* < 0.01, *** *p* < 0.001, **** *p* < 0.0001, *p* > 0.05; ns, not significant. **(D)** Design of OPNAs introducing a G-clamp modification. The structure of the G-clamp is shown with the modified residue in red.

Since OPNAs targeting the 5’ UTR region, including 530, 531, 533, and 536, showed good antiviral activity (Figure 2E), based on them, we designed modified OPNAs to investigate their antiviral ability. According to the data presented in Figure 5A, extending the length of OPNA 530 by 4mer, or adjusting the position of the modification, did not improve the inhibitory impact of the OPNA as both OPNA 530 and 549 had almost similar effects (Figure 5A). In contrast, the corresponding effects of the modified OPNA 531 (OPNA 566) suppressed viral expression more dramatically with approximately 25% more potency. This improved performance was most likely related to the substitution of A* with G* at the 3′ end of the OPNA oligomer (Figure 5B). Regarding the OPNA 533, altering the length and number of modifications resulted in a preferable antiviral effect which was comparable to the inhibitory level of OPNA 11 (~50%) (Figure 5C). Unexpectedly, however, introducing the G-clamp modification (C**), which is known to enhance specificity and thermal stability of the formed duplex structure (Rajeev et al., 2002), rather reduced the viral suppressive activity of OPNA 536 dramatically (Figure 5D). Collectively, by adjusting different elements of modification, the most effective OPNA targeting the 5’ UTR region was discovered.

### 3.6. Modified OPNA targeting pseudoknot slippery site interfered with HCoV-OC43 replication

**Figure 6:**
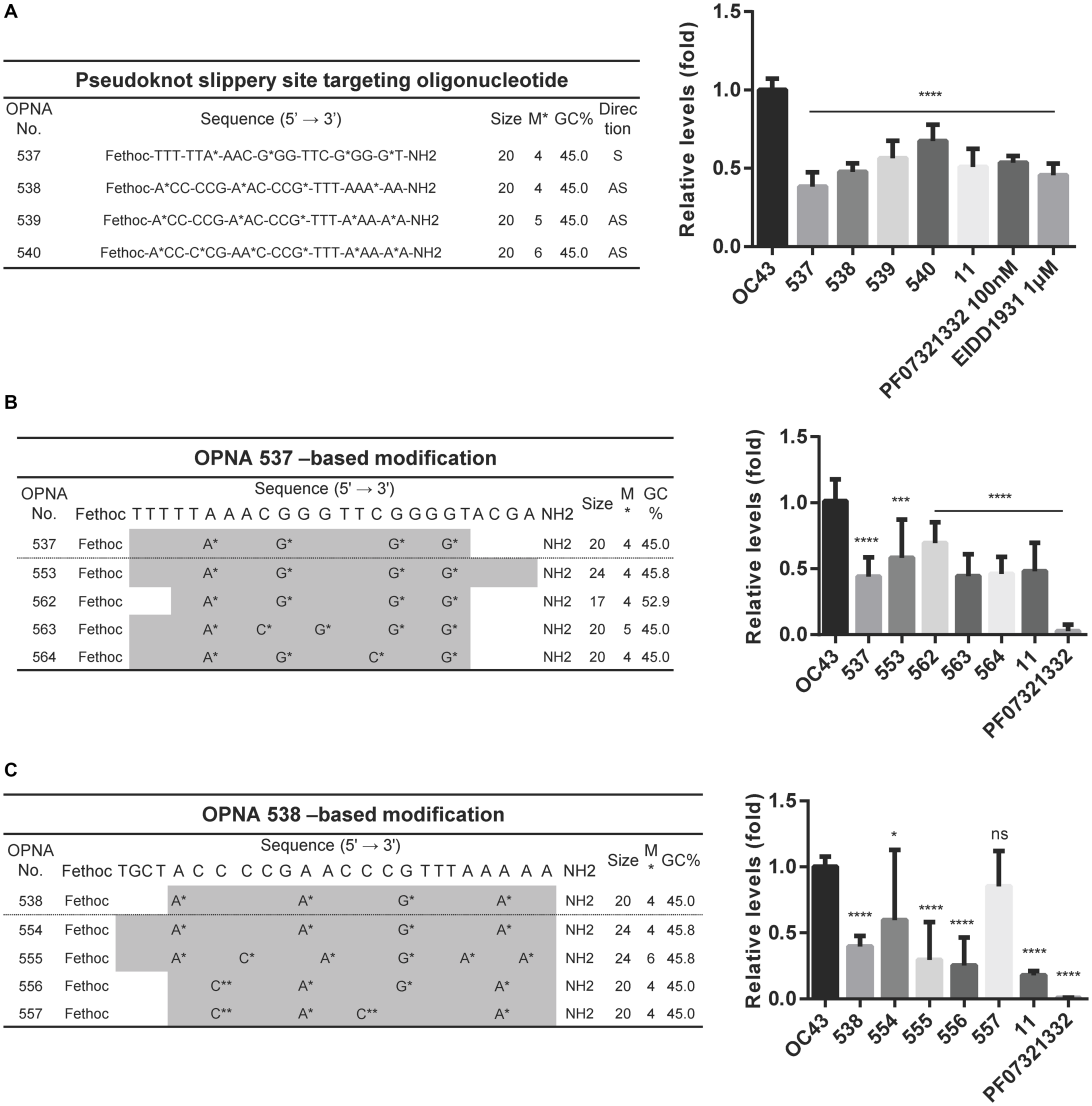
Decreasing the HCoV-OC43 expression by modified OPNAs targeting pseudoknot and slippery sites. **(A)** OPNAs targeting pseudoknot and slippery sites were designed and tested for antiviral effects. **(B,C)** OPNA 537- and 538-based modification and inhibition of viral replication. The OPNA sequences are marked in gray. For **(A–C)**, HCT-8 cells were infected with HCoV-OC43 virus at 500 TCID_50_, and OPNAs were treated at 1 μM in a 6-well plate. Cell supernatant was collected at 3 dpi. Data were presented as means ± SEM of three independent experiments, and Student’s *t*-test was used to determine the significance. OC43 (control) versus OPNA-treated group in each experiment, symbols represent mean ± SD. ** *p* < 0.01, *** *p* < 0.001, **** *p* < 0.0001, *p* > 0.05; ns, not significant.

The OPNA 7, an antisense OPNA oligomer, targeting the pseudoknot slippery site and OPNA 506, a sense OPNA oligomer, targeting the same region were developed and examined for antiviral efficiency in Figures 2A, 3A, respectively. Nonetheless, virus reduction levels were not observed. One reason might be that the 14-mer length of these OPNA oligomers was too short for the 20mer that was once thought to be the ideal size for function. We, therefore, sought to design a sense and three antisense oligos specific to different positions within the pseudoknot slippery site while maintaining the oligo size at 20-mer. Although the 537 to 540 oligos were able to reduce the OC43 levels, there seemed to be no benefit to target the other sites, with OPNA 537 having the greatest inhibitory effect on viral titers (Figure 6A). Consequently, OPNA 537 was subjected to a combination of length and modification adjustments, and the *in vitro* antiviral effect of these oligos was assessed (Figures 6B,C). The RT-qPCR results indicated that the virus inhibitory effect of OPNA 563 and 564 was similar to that of 537, with an almost 55% reduction (Figure 6B). Finally, among the OPNA 538-based modifications, OPNA 556 represented the most promising results with higher potency compared with the template oligo 538 (Figure 6C). This enhanced antiviral activity was achieved by incorporating the G-clamp (Rajeev et al., 2002; Ortega et al., 2007) (C**) structure, as shown in Figures 5D, 6C. Taken together, the above results suggest that pseudoknot and slippery sites are good candidate targets for ASO and that effective antisense OPNA oligos can be obtained by controlling the length and base modification of OPNAs, targeting these sites.

### 3.7. Antiviral activity of selected OPNAs by time-of-addition assay

**Figure 7:**
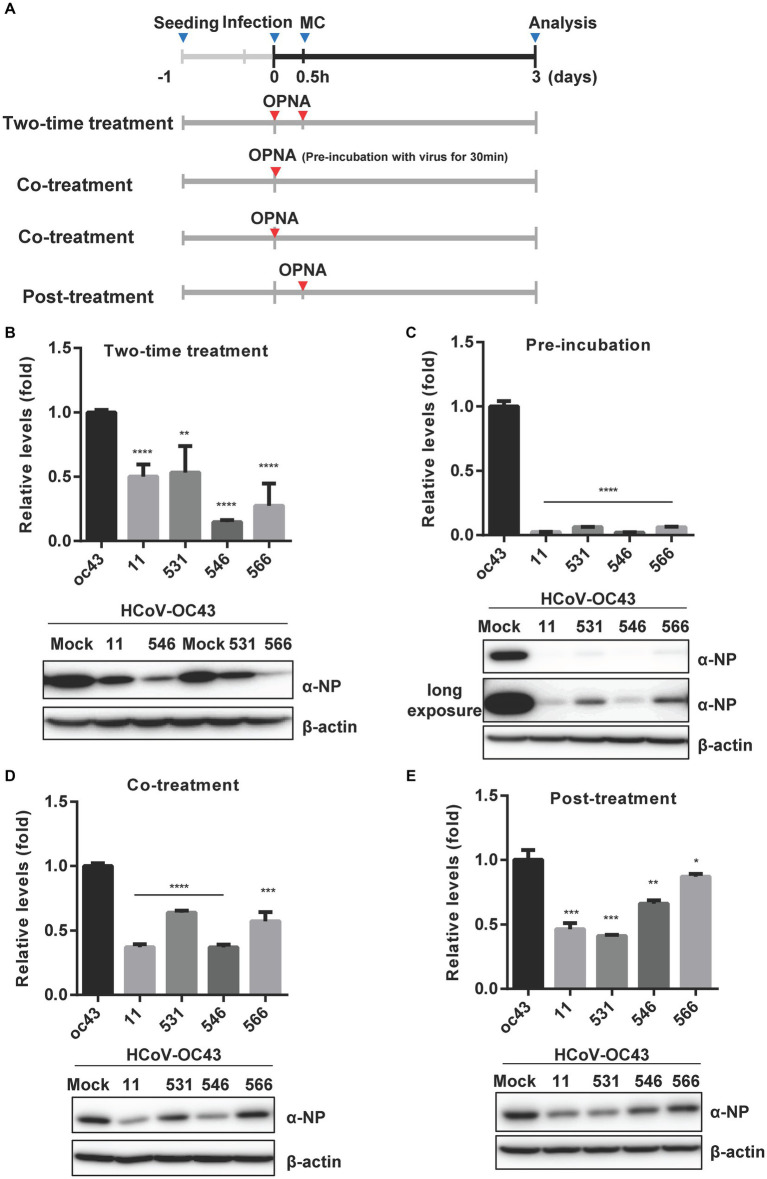
Time-of-addition assay of selected OPNAs. **(A)** Scheme of time-of-addition assay in HCT-8 cells. **(B)** Antiviral activity of OPNAs by two-time treatment method. **(C)** Inhibition of viral replication of OPNAs by pre-incubation with virus for 30 min. **(D)** Antiviral activity of OPNAs by co-treatment method. **(E)** Antiviral activity of OPNAs by post-treatment method. For **(B–E)**, HCT-8 cells were infected with HCoV-OC43 virus at 500 TCID_50_, and OPNAs were treated at 1 μM in a 6-well plate. Cell supernatant was collected at 3 dpi. Viral titration was performed by RT-qPCR. For Western blot, the NP antibody (HCoV-OC43) was used at a 1:2000 dilution ratio. β-actin was used as a loading control. Data were presented as means ± SEM of three independent experiments, and Student’s *t*-test was used to determine the significance. OC43 (control) versus OPNA-treated group in each experiment, symbols represent mean ± SD. ** *p* < 0.01, *** *p* < 0.001, **** *p* < 0.0001, *p* > 0.05; ns, not significant.

Time-of-addition assay (Min et al., 2020) was performed to determine and compare the effect of four selected OPNAs in HCT-8 cells. The experiments were conducted with four treatment methods along with 30 min virus infection scheme (Figure 7A). Using the two-time treatment approach, only 2 OPNA oligomers (11 and 546) showed noticeable antiviral activity (Figure 7B). Incubation of virus and OPNA in one tube for 30 min prior to infection resulted in a strong decrease in HCoV-OC43 and inhibited the expression of the nucleoprotein (NP), which was confirmed by RT-qPCR and Western blot, respectively. After 3 days of infection, the virus titer and NP protein levels in OPNA 11, 531, 546, and 566-treated cells drastically decreased, nearly eliminating viral replication (Figure 7C). Unlike the two-time treatment strategy with the 531 and 566, which failed to significantly reduce virus titer, co-treating these OPNA oligomers with virus at the time of infection proved effective (Figure 7D), and more interestingly, the OPNA 531 exhibited the highest antiviral activity among the other four oligos through post-treatment (Figure 7E). Overall, the pre-incubation treatment strategy proved to be the most efficient method in our system for significantly lowering viral titer and NP protein production.

### 3.8. Determination of IC_50_ and translational inhibition of viral protein by selected OPNAs

**Figure 8:**
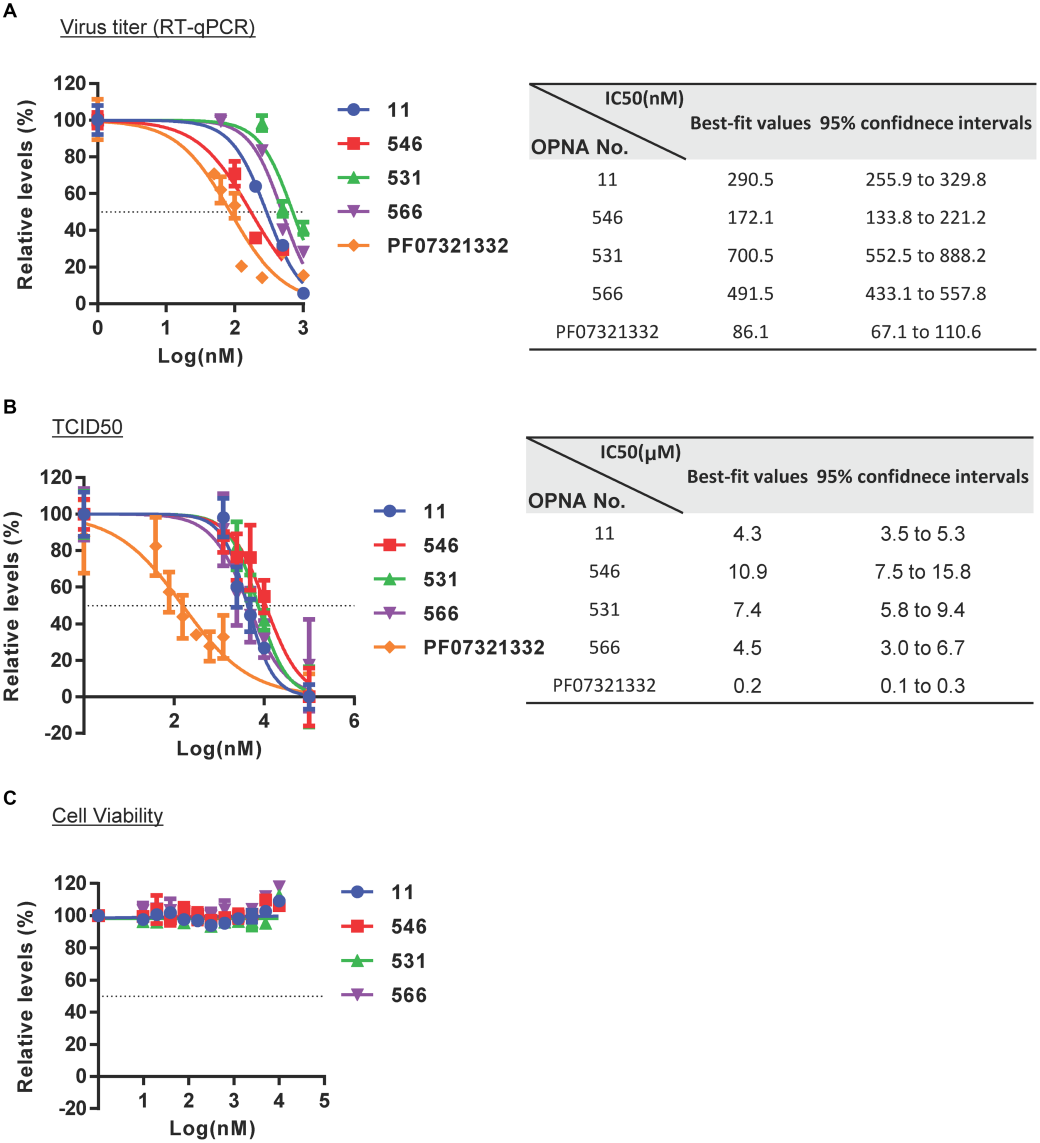

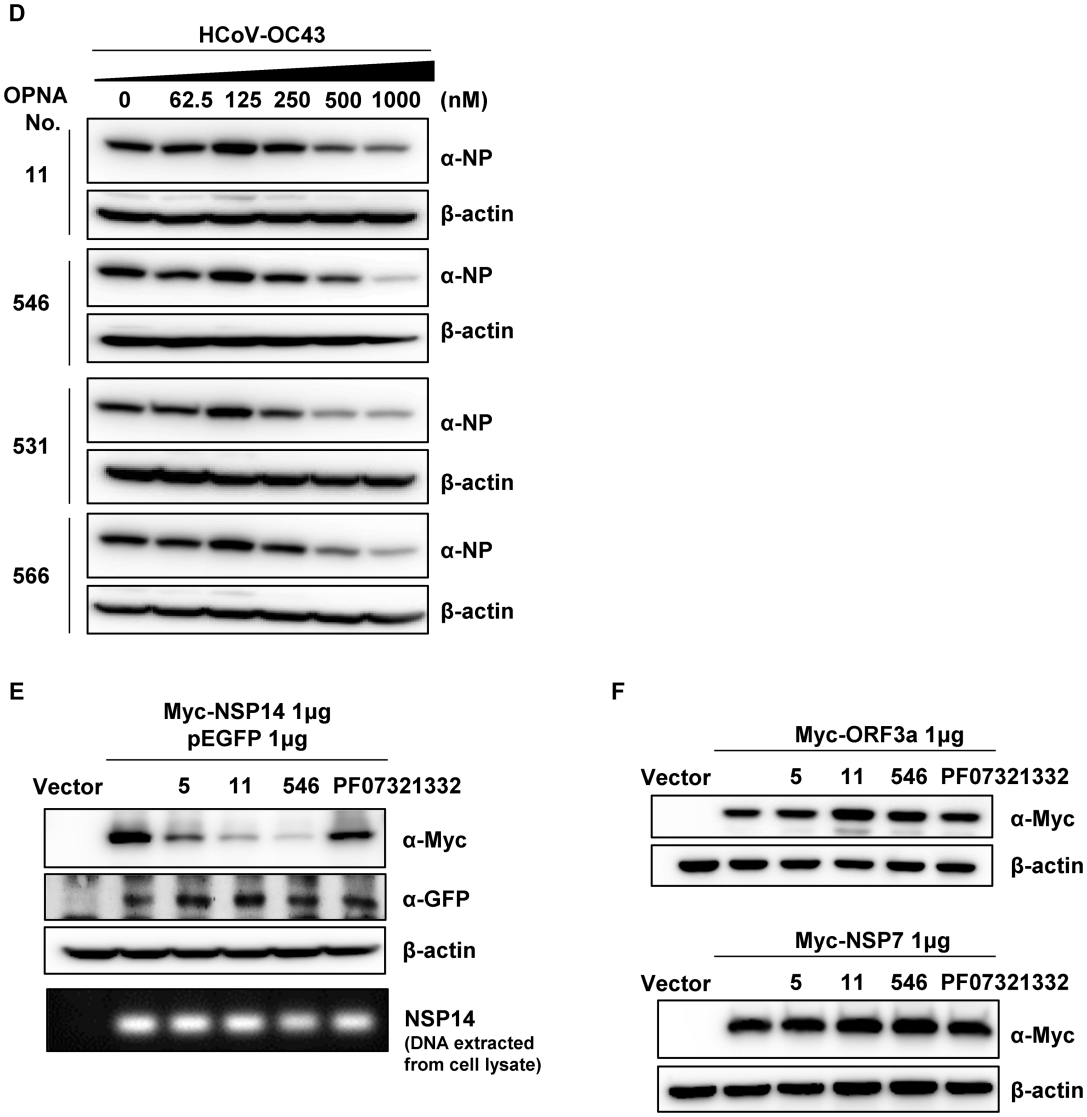
IC_50_ determination and reduction of NSP14 protein levels by selected OPNAs. **(A)** Determination of IC_50_ values based on virus titers measured by RT-qPCR. Viral RNAs were isolated from the supernatant of infected HCoV-OC43 cells. **(B)** Supernatant of IC_50_ values by TCID_50_ using Viral ToxGlo Assay Kit. IC_50_ values were determined using a Viral ToxGlo Assay Kit after infection with HCoV-OC43 at 300 TCID_50_ in a 96-well plate. The experiments were performed at 5 dpi. **(C)** Cell viability assay by CellTiter-Glo^®^ Luminescent Cell Viability Assay Kit on the second day following treatment. **(D)** Western blot assay for viral NP of HCoV-OC43 after OPNA treatment. Cells were harvested at 3 dpi with 500 TCID_50_ infections in a 6-well for Western Blot analysis. **(E)** Translational inhibition of SARS-CoV-2 NSP14 protein by OPNA treatment. Protein levels were detected by Western blot using anti-Myc and anti-GFP antibodies. Protein levels of GFP and NSP14 DNA extracted from cell lysate were provided as transfection control. **(F)** Target-specific assay for OPNA. SARS-CoV-2 ORF3a and NSP7 do not contain binding sequences with OPNAs 5, 11, and 546.

Following the identification of four potent OPNAs that effectively inhibit HCoV-OC43 titer and its NP protein expression, we measured their IC_50_ levels according to the virus titer and TCID_50_ methods, respectively (Figures 8A,B). To determine the IC_50_ of selected OPNA oligomers, they were treated to HCoV-OC43-infected cells in a dose-dependent manner along with PF07321332. Three days post-infection, the cell supernatant was collected to analyze virus titer by RT-qPCR. The IC_50_ values for OPNA 11, 546, 531, 566, and PF07321332 were 290.5 nM, 172.1 nM, 700.5 nM, 491.5 nM, and 86.1 nM, respectively (Figure 8A). Meanwhile, the TCID_50_ was measured according to the CPE caused by the virus at 5 days after infection. The TCID_50_ levels are presented in Figure 8B. Based on the TCID_50_, the calculated IC_50_ values were 4.31 μM for OPNA 11, 10.86 μM for OPNA 546, 7.36 μM for OPNA 531, 4.50 μM for OPNA 566, and 0.16 μM for PF07321332 (Figure 8B). For IC_50_ determination, the TCID_50_ method was much more sensitive than that of RT-qPCR. In addition, we confirmed the cell viability of OPNA using the CellTiter-Glo^®^ Luminescent Cell Viability Assay Kit, and OPNAs exhibited no cell toxicity as shown in Figure 8C.

In addition, NP protein levels were measured using Western blotting to ascertain whether OPNA treatment reduced viral protein levels. As shown in Figure 8D, escalating doses of OPNA substantially decreased NP protein levels, particularly above 250 nm.

As the OPNAs were designed to bind to the SARS-CoV-2 NSP14 sequence, their specificity was evaluated by measuring the NSP14 protein level after ectopic expression of the Myc-NSP14 construct (Figure 8E). Additionally, pEGFP was used as the transfection control. The transfected DNA was isolated using TRI reagent, and the NSP14 gene levels were determined using semi-quantitative PCR (Figure 8E). As predicted, the expression of NSP14 protein was significantly reduced in samples treated with OPNA 5, 11, and 546 compared with untreated or PF07321332-treated samples, suggesting that OPNAs inhibit the transcription or translation of target genes. In addition, we confirmed that OPNA 11 had no effect on other SARS-CoV-2 genes that do not contain the target sequence of OPNA11 (Figure 8F). These results indicate that, similar to the general mechanism of ASO, OPNAs bind specifically to SARS-CoV-2 NSP14 gene or RNA and inhibit translation.

### 3.9. The combination of OPNA with different targets reveals greater activity against HCoV-OC43 and SARS-CoV-2

**Figure 9:**
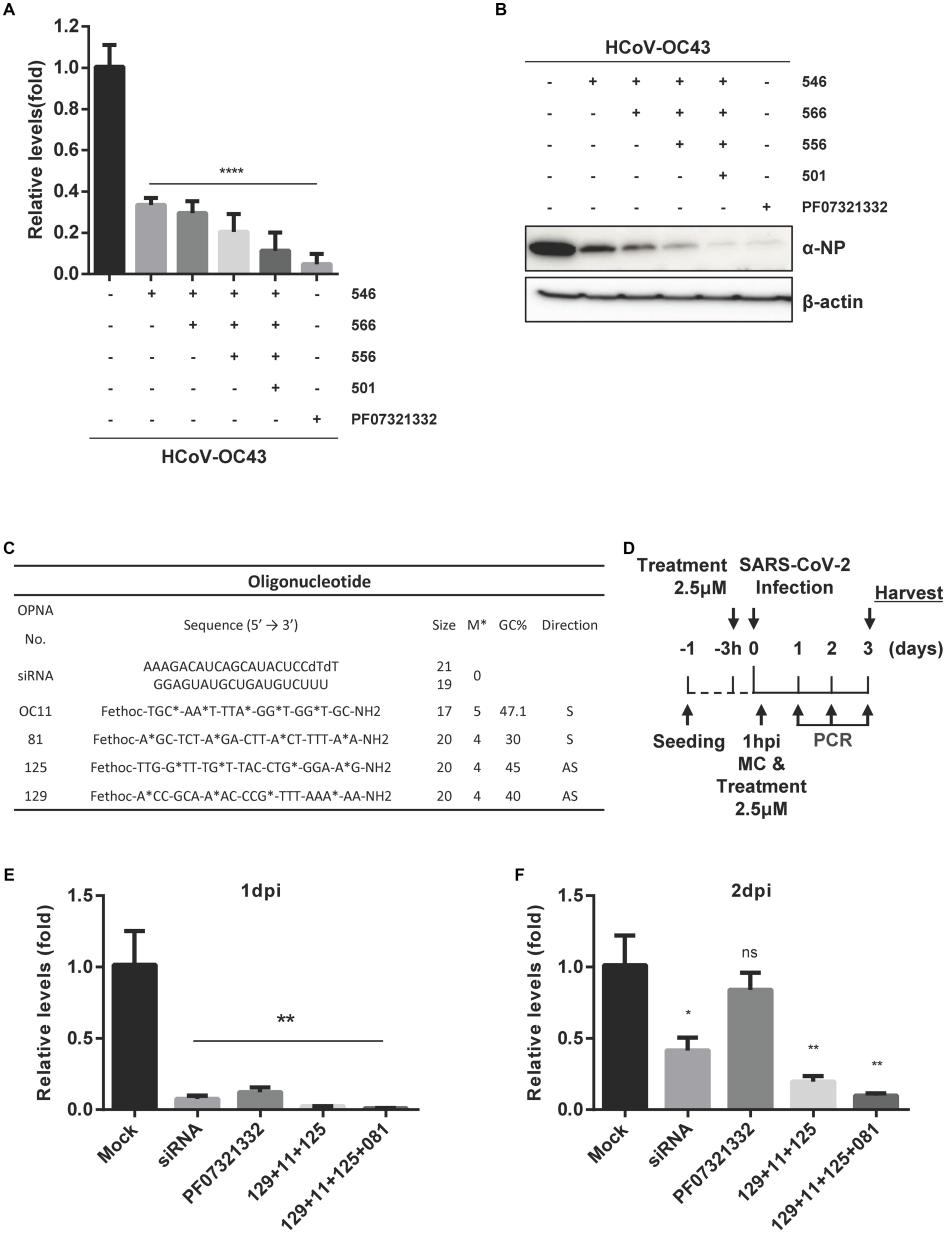

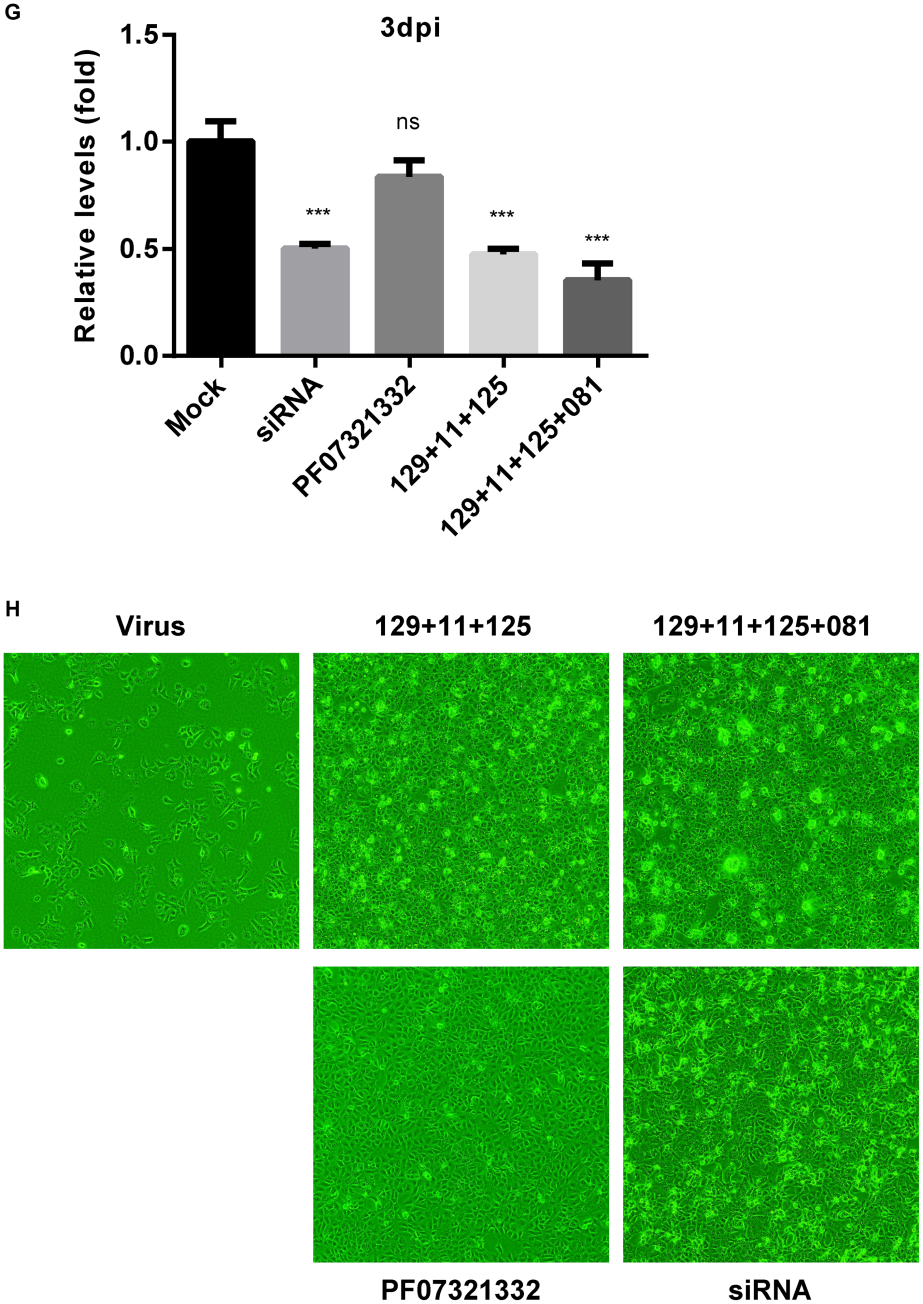
Inhibitory effect of a combination of OPNAs targeting different conserved coronavirus regions. **(A)** The viral inhibitory effect of a combination of OPNAs was confirmed by viral titer in the HCoV-OC43 infection system. OPNAs and PF07321332 were treated at 1 μM. Viral RNAs were isolated from the supernatant of HCT-8 cells. Viral titers were determined by RT-qPCR. **(B)** The inhibitory effect of a combination of OPNAs on viral NP protein levels was determined by Western blot. **(C)** Design of OPNAs targeting SARS-CoV-2. **(D)** Experimental scheme to assess the antiviral efficacy of OPNAs against SARS-CoV-2 in Vero E6 cells. Hpi, hour post-infection; MC, media change. **(E–G)** The antiviral activity of OPNAs was tested by measuring viral titers in SARS-CoV-2-infected cells. Viral RNAs were isolated from cell supernatant of SARS-CoV-2-infected cells. Cells were infected at 250 PFU/well in a 24-well plate. Titration was performed by RT-qPCR. Cell supernatants were collected in 140 μL at 1, 2, and 3 dpi for each sample. **(H)** Confirmation of CPE improvement by OPNA treatment after SARS-CoV-2 infection in Vero E6 cells. Data were presented as means ± SEM of three independent experiments, and Student’s *t*-test was used to determine the significance. Mock (virus infection only) versus OPNA-treated group in each experiment, symbols represent mean ± SD. ** *p* < 0.01, *** *p* < 0.001, **** *p* < 0.0001, *p* > 0.05; ns, not significant.

Next, the antiviral ability of a combination of OPNAs targeting multiple sites was examined when treated simultaneously. Experiments were conducted by treating 1 μM of OPNA 566 (targeting the 5’ UTR of HCoV-OC43 virus), OPNA 546 (targeting NSP14), OPNA 556 (targeting pseudoknot slippery site), and OPNA 501 (targeting RdRp) (Figures 9A,B). When the infected cells were treated with a mixture of oligos, the virus titer and NP protein levels decreased dramatically by more than 8-fold.

To demonstrate that the designated OPNAs are applicable to SARS-CoV-2 (Figure 9C), as the final stage of oligo screening in our study, a mixture of OPNAs targeting the sites similar to that of HCoV-OC43 was synthesized and treated 3 h before infection and 1 h after infection, and the viral titers in the culture supernatant were measured daily from 1 to 3 dpi (Figure 9D). The maximum antiviral effect was observed 1 day after infection. Similar outcomes were observed when a siRNA specific for SARS-CoV-2 was applied (Figures 9E–G). The treatment with a viral 3CL protease inhibitor did not inhibit viral titers at 2 or 3 dpi, whereas OPNA had a significantly greater anti-SARS-CoV-2 effect (Owen et al., 2021). Of note, the cells were not treated with efflux inhibitor in this experiment. Eventually, the immunofluorescence data presented in Figure 9H clearly demonstrated that the combination of OPNAs targeting multiple regions significantly reduced CPE 3 days after SARS-CoV-2 infection, demonstrating their effectiveness in controlling viral infection.

## 4. Discussion

The COVID-19 pandemic has had negative effects, resulting not only in significant health-related complications but also in numerous negative effects on the global economy and society.

To combat the current outbreak more effectively and to be prepared for the potential emergence of new respiratory viruses, a treatment platform that can be quickly implemented and adapted for large-scale production is required. One of the potential benefits of ASOs as potential drug candidates is that they can be designed to target only the RNA molecules that are implicated in the pathogenesis of diseases. However, there are a few obstacles associated with the development of ASO medications, including the need to optimize their pharmacokinetic properties for efficient delivery to the target tissues. By resolving these issues, ASOs would become a promising area of drug development with important applications in the treatment of a wide range of diseases.

Up to now, a number of FDA-approved ASO medications, including fomivirsen, mipomersen, inotersen, nusinersen, and patisiran, have been released onto the market. In addition, more nucleic acid-based pharmaceuticals are presently undergoing different phases of clinical trials, and some are being researched for the treatment of various diseases (Roberts et al., 2020).

PNA-based ASOs have been deemed as attractive drug candidates, but intracellular delivery has been a substantial hurdle. Here, we present modified PNAs, OPNAs, with exceptional cell permeability that disrupt different important conserved regions of the SARS-CoV-2 and HCoV-OC43 genomes. Interestingly, we aligned the sequences related to the OPNA11 and SARS-CoV-2 variants, and the conserved sequences were matched 100% accurately with SARS-CoV and SARS-CoV-2-Wuhan strains, as well as delta and omicron variants. The ability of chemically modified OPNA to cross cell membranes suggests that it could be rapidly delivered directly to the nasal cavity without a carrier in a future pandemic. In addition to their highly enhanced permeability, OPNAs can also be used to regulate pre-mRNA splicing because they can bind RNA at much lower concentrations than other types of artificial nucleic acids and prevent the complex formation of spliceosome (Chung et al., 2017).

In this study, we designed and screened PNA ASOs capable of modulating HCoV-OC43 and SARS-CoV-2 infection. In our *in vitro* experiments, OPNA 546 inhibited HCoV-OC43 infection with an IC_50_ of 133.8–221.2 nM (Figure 8A). Further optimizations of binding affinity and cell permeability are being performed to reduce the IC_50_ value to less than 100 nM, which is optimal for drug production. The antiviral activity of Pfizer’s protease inhibitor was greater than ours. However, this compound was utilized to validate the authenticity of our own method rather than for the purpose of comparing efficacy.

Our observation, here, indicated that various OPNAs with different sizes and numbers of modifications exhibit a range of activity. Interestingly, we did not observe any specific pattern or dose dependency in the activity of the designed oligonucleotides. This lack of consistency in activity could be attributed to the inherent differences in sequence-specific binding affinity and penetration efficacy caused by the secondary structures of viral RNA itself. RNA possesses complex secondary structures that can influence the targeting abilities of the oligonucleotides.

Notably, pre-incubation of oligos with the virus significantly increased their inhibitory effect on the virus titer and protein expression level (Figure 7C), indicating that OPNA interferes with the virus during the early stages of infection, possibly by entering the virus particle and attaching to the virus genome or by binding the virus when its genome is exposed during cell infection. More comprehensive mechanistic studies are needed to fully elucidate the interaction with the pathogen.

Moreover, as shown in Figures 8D,E, the protein level of GFP, Myc-ORF3a, and Myc-NSP7 was unaffected by OPNA treatment, whereas Myc-NSP14 protein levels decreased, suggesting that OPNAs inhibit protein production by binding to target specifically. Importantly, our study revealed that a combination of oligos inhibited the virus more effectively (Figures 9A,B).

In conclusion, we have discovered a novel strategy that modified PNA ASOs targeting common conserved regions of SARS-CoV-2 and HCoV-OC43 and inhibited the progression of coronavirus infection, making them promising candidates for a new therapeutic approach against COVID-19 and the next COVID. Our studies indicate that OPNAs may be a promising platform for the development of antivirals against future pandemic viral infections that do not require a delivery vehicle.

## Data availability statement

The datasets presented in this study can be found in online repositories. The names of the repository/repositories and accession number(s) can be found in the article/Supplementary material.

## Author contributions

SP: Conceptualization, Investigation, Writing – review & editing, Data curation, Formal analysis, Methodology, Project administration, Validation, Visualization, Writing – original draft. SHK: Data curation, Investigation, Writing – original draft. MD: Writing – original draft, Writing – review & editing. E-HL: Writing – original draft, Investigation. YJ: Investigation, Resources, Validation, Writing – original draft, Writing – review & editing. DJ: Resources, Project administration, Writing – original draft, Writing – review & editing. SG: Funding acquisition, Investigation, Writing – original draft. CY: Funding acquisition, Investigation, Writing – original draft. SL: Funding acquisition, Investigation, Resources, Writing – original draft. SCK: Funding acquisition, Resources, Project administration, Writing – review & editing. K-HK: Funding acquisition, Conceptualization, Investigation, Supervision, Writing – original draft, Writing – review & editing.

## Funding

The author(s) declare financial support was received for the research, authorship, and/or publication of this article. This study was supported by the National Research Foundation of Korea (NRF) grants funded by the Korea Government [NRF- 2021M3A912080488] and a grant from the Korea Health Technology R&D Project through the Korea Health Industry Development Institute (KHIDI), funded by the Ministry of Health & Welfare, Republic of Korea (grant number: HI23C0673). This research was also funded by the Agency for Defense Development, Republic of Korea (grant numbers UE211116ZD and 311KK5-912888601).

## Conflict of interest

YJ, DJ, and SCK were employed by OliPass Corporation.

The remaining authors declare that the research was conducted in the absence of any commercial or financial relationships that could be construed as a potential conflict of interest.

## Publisher’s note

All claims expressed in this article are solely those of the authors and do not necessarily represent those of their affiliated organizations, or those of the publisher, the editors and the reviewers. Any product that may be evaluated in this article, or claim that may be made by its manufacturer, is not guaranteed or endorsed by the publisher.
