# Supplementary material for: Cell-permeable peptide nucleic acid antisense oligonucleotide platform targeting human betacoronaviruses
Source: Front Microbiol. 2023 Sep 29;14:1258091. doi: 10.3389/fmicb.2023.1258091 (PMC10570754; doi:10.3389/fmicb.2023.1258091)

Supplementary Table 1. Total oligomer information

Name	Sequence (N → C)	Size	M*	GC%	Target Locations
1	Fethoc-A(5)TA-G(6)CA-GC(12)A-TTA(5)-CCA(5)-TCC(12)-TG-NH2	20	6	45.0	14770-14789/SARS-CoV-2, HCoV-OC43
2	Fethoc-G(6)GA-TA(5)A-TC(12)C-CA(5)A-CC(12)C-ATA(5)-AG-NH2	20	6	45.0	15280-15299/SARS-CoV-2, HCoV-OC43
3	Fethoc-CA(5)C-TC(12)T-AG(6)T-G(6)TC-AA(5)A-TC(12)T-AC-NH2	20	6	40.0	19235-19254/SARS-CoV-2, HCoV-OC43
4	Fethoc-G(6)TG-TG(6)G-AA(5)T-GC(12)A-TG(6)T-TTA(5)-TT-NH2	20	6	35.0	19303-19322/SARS-CoV-2, HCoV-OC43
5	Fethoc-GCA(5)-CCA(5)-CC(12)T-AAA(5)-TTG(6)-CA-NH2	17	5	47.1	19468-19484/SARS-CoV-2, HCoV-OC43
6	Fethoc-TTA(5)-CAC(12)-CAA(5)-AGC(12)-ATA(5)-AA-NH2	17	5	29.4	20605-20621/SARS-CoV-2, HCoV-OC43
7	Fethoc-A(5)AC(12)-CCG(6)-TTT-A(5)AA-A(5)A-NH2	14	5	35.3	13460-13473/SARS-CoV-2, HCoV-OC43
8	Fethoc-A(5)CA-TC(12)T-G(6)AA-TC(12)A-A(5)-NH2	13	5	30.8	8190-8202/SARS-CoV-2, HCoV-OC43
9	Fethoc-AG(6)T-TA(5)T-TA(5)C-A(5)AC(12)-T-NH2	13	5	23.1	8270-8282/SARS-CoV-2, HCoV-OC43
10	Fethoc-G(6)TA-A(5)TA-G(6)CA-A(5)CA(5)-TT-NH2	14	5	28.6	17920-17933/SARS-CoV-2, HCoV-OC43
11	Fethoc-TGC(12)-AA(5)T-TTA(5)-GG(6)T-GG(6)T-GC-NH2	17	5	47.1	19468-19484/SARS-CoV-2, HCoV-OC43
501	Fethoc-C(12)AG-G(6)AT-G(6)GT-A(5)AT-GC(12)T-GCT-A(5)T-NH2	20	6	45.0	14770-14789/SARS-CoV-2, HCoV-OC43
502	Fethoc-C(12)TT-A(5)TG-G(6)GT-TG(6)G-GA(5)T-TAT-C(12)C-NH2	20	6	45.0	15280-15299/SARS-CoV-2, HCoV-OC43
503	Fethoc-G(6)TA-GA(5)T-TTG(6)-AC(12)A-CTA(5)-GAG(6)-TG-NH2	20	6	40.0	19235-19254/SARS-CoV-2, HCoV-OC43
504	Fethoc-A(5)AT-A(5)AA-C(12)AT-G(6)CA-TTC(12)-CAC-A(5)C-NH2	20	6	35.0	19303-19322/SARS-CoV-2, HCoV-OC43
505	Fethoc-TTT-A(5)TG-C(12)TT-TG(6)G-TG(6)T-AA(5)-NH2	17	5	29.4	20605-20621/SARS-CoV-2, HCoV-OC43
506	Fethoc-TTT-TTA(5)-AA(5)C-G(6)GG(6)-TT-NH2	14	4	35.3	13460-13473/SARS-CoV-2, HCoV-OC43
507	Fethoc-TTG-A(5)TT-C(12)AG-A(5)TG(6)-T-NH2	13	4	30.8	8190-8202/SARS-CoV-2, HCoV-OC43
508	Fethoc-AG(6)T-TG(6)T-AA(5)T-AA(5)C-T-NH2	13	4	23.1	8270-8282/SARS-CoV-2, HCoV-OC43
509	Fethoc-A(5)AT-G(6)TT-G(6)CT-A(5)TT-A(5)C-NH2	14	5	28.6	17920-17933/SARS-CoV-2, HCoV-OC43
510	Fethoc-TGC-AAT-TTA-GGT-GGT-GC-NH2	17	0	47.1	19468-19484/SARS-CoV-2, HCoV-OC43
511	Fethoc-TGC-AA(5)T-TTA(5)-GG(6)T-GG(6)T-GC-NH2	17	4	47.1	19468-19484/SARS-CoV-2, HCoV-OC43
512	Fethoc-TG(6)C-AA(5)T-TTA(5)-GG(6)T-GG(6)T-GC-NH2	17	5	47.1	19468-19484/SARS-CoV-2, HCoV-OC43
513	Fethoc-TG(6)C-AA(5)T-TTA-G(6)GT-G(6)GT-G(6)C-NH2	17	5	47.1	19468-19484/SARS-CoV-2, HCoV-OC43
514	Fethoc-AGA(5)-TGC(12)-AA(5)T-TTA(5)-GG(6)T-GG(6)T-GC-NH2	20	6	45.0	19316-19335/HCoV-OC43
515	Fethoc-TGC(12)-AA(5)T-TTA(5)-GG(6)T-GG(6)T-GCA(5)-GT-NH2	20	6	45.0	19319-19338/HCoV-OC43
516	Fethoc-TGC(12)-AA(5)T-TTA(5)-GG(6)-NH2	14	4	42.9	19319-19332/SARS-CoV-2, HCoV-OC43
517	Fethoc-C(12)AA(5)T-TTT-A(5)GG(6)-TGG(6)-TG-NH2	14	5	42.9	19321-19334/SARS-CoV-2, HCoV-OC43
518	Fethoc-TGC-AA(5)T-TTA-GGT-GGT-GC-NH2	17	1	47.1	19468-19484/SARS-CoV-2, HCoV-OC43
519	Fethoc-TGC-AAT-TTA(5)-GGT-GGT-GC-NH2	17	1	47.1	19468-19484/SARS-CoV-2, HCoV-OC43
520	Fethoc-TGC-AA(5)T-TTA(5)-GGT-GGT-GC-NH2	17	2	47.1	19468-19484/SARS-CoV-2, HCoV-OC43
521	Acetyl-TGC-AAT-TTA-GGT-GGT-GC-NH2	17	0	47.1	19468-19484/SARS-CoV-2, HCoV-OC43
522	Acetyl-TGC-AA(5)T-TTA-GGT-GGT-GC-NH2	17	1	47.1	19468-19484/SARS-CoV-2, HCoV-OC43
523	Acetyl-TGC-AAT-TTA(5)-GGT-GGT-GC-NH2	17	1	47.1	19468-19484/SARS-CoV-2, HCoV-OC43
524	Acetyl-TGC-AA(5)T-TTA(5)-GGT-GGT-GC-NH2	17	2	47.1	19468-19484/SARS-CoV-2, HCoV-OC43
525	Acetyl-TGC(12)-AA(5)T-TTA(5)-GG(6)T-GG(6)T-GC-NH2	17	5	47.1	19468-19484/SARS-CoV-2, HCoV-OC43
526	H-TGC(12)-AA(5)T-TTA(5)-GG(6)T-GG(6)T-GC-NH2	17	5	47.1	19468-19484/SARS-CoV-2, HCoV-OC43
527	Fethoc-GCA-CCA(5)-CCT-A(5)AA-TTG-CA-NH2	17	2	47.1	19468-19484/SARS-CoV-2, HCoV-OC43
528	Fethoc-GCA(5)-CCA(5)-CCT-A(5)AA-TTG-CA-NH2	17	3	47.1	19468-19484/SARS-CoV-2, HCoV-OC43
529	Fethoc-GCA(5)-CGC-AA(5)A-TCG(6)-CTC-ACA(5)-AT-NH2	20	4	50.0	1-20/HCoV-OC43
530	Fethoc-TGA-A(5)GC-GGG(6)-ATG-CA(5)C-GCA(5)-CG-NH2	20	4	65.0	16-35/HCoV-OC43
531	Fethoc-CTA(5)-ACA-A(5)GA-GAT-CA(5)G-TGA-A(5)G-NH2	20	4	51.3	31-50/HCoV-OC43
532	Fethoc-AGA(5)-TTA-CAA(5)-AAA-GA(5)T-CTA-A(5)C-NH2	20	4	25.0	46-65/HCoV-OC43
533	Fethoc-GTT-TA(5)G-ATT-A(5)CA-AA(5)A-AGA(5)-TC-NH2	20	4	25.0	50-69/HCoV-OC43
534	Fethoc-G(6)TT-TTT-A(5)TA-AA(5)G-TTT-A(5)GA-TT-NH2	20	4	41.1	61-80/HCoV-OC43
535	Fethoc-TTA(5)-CA(5)G-GGA(5)-GTG-GA(5)T-GTT-TT-NH2	20	4	40.0	76-95/HCoV-OC43
536	Fethoc-G(6)CC-CAC-A(5)AG-CAT-A(5)GA-TTA(5)-CA-NH2	20	4	45.0	91-110/HCoV-OC43
537	Fethoc-TTT-TTA(5)-AAC-G(6)GG-TTC-G(6)GG-G(6)T-NH2	20	4	45.0	13332-13351/ HCoV-OC43
538	Fethoc-A(5)CC-CCG-A(5)AC-CCG(6)-TTT-AAA(5)-AA-NH2	20	4	45.0	13332-13351/ HCoV-OC43
539	Fethoc-A(5)CC-CCG-A(5)AC-CCG(6)-TTT-A(5)AA-A(5)A-NH2	20	5	45.0	13332-13351/ HCoV-OC43
540	Fethoc-A(5)CC-C(12)CG-AA(5)C-CCG(6)-TTT-A(5)AA-A(5)A-NH2	20	6	45.0	13332-13351/ HCoV-OC43
541	Benzoyl-TGC(12)-AA(5)T-TTA(5)-GG(6)T-GG(6)T-GC-NH2	17	5	47.1	19468-19484/SARS-CoV-2, HCoV-OC43
544	Fethoc-TGC(Ph2)-AA(5)T-TTA(5)-GG(6)T-GG(6)T-GC-NH2	17	5	47.1	19468-19484/SARS-CoV-2, HCoV-OC43
545	Fethoc-CAA-GA(5)T-GC(12)A-A(5)TT-TA(5)G-G(6)TG-G(6)TG-C-NH2	22	6	45.5	19314-19335/HCoV-OC43
546	Fethoc-CA(5)C-AAG-A(5)TG-C(12)AA(5)-TTT-A(5)GG(6)-TGG(6)-TGC-NH2	24	7	45.8	19312-19335/HCoV-OC43
547	Fethoc-ATC-A(5)CA-AGA(5)-TGC(12)-AA(5)T-TTA(5)-GG(6)T-GG(6)T-GC-NH2	26	7	42.3	19310-19335/HCoV-OC43
548	Fethoc-ATC-A(5)CA-AGA(5)-TGC-AA(5)T-TTA(5)-GGT-G(6)GT-GC-NH2	26	5	42.3	19310-19335/HCoV-OC43
549	Fethoc-TCA-GTG-AA(5)G-CGG(6)-GAT-GCA(5)-CGC-A(5)CG-NH2	24	4	62.5	16-39/HCoV-OC43
550	Fethoc-TCA(5)-GTG-AA(5)G-CGG(6)-GAT-GCA(5)-CGC-A(5)CG-NH2	24	5	62.5	16-39/HCoV-OC43
551	Fethoc-AGA(5)-TCT-A(5)AC-AA(5)G-AGA-TCA(5)-GTG-AA(5)G-NH2	24	5	37.5	31-54/HCoV-OC43
552	Fethoc-GTT-TA(5)G-ATT-A(5)CA-AA(5)A-AGA(5)-TCT-AA(5)C-NH2	24	5	25.0	46-69/HCoV-OC43
553	Fethoc-TTT-TTA(5)-AAC-G(6)GG-TTC-G(6)GG-G(6)TA-CGA-NH2	24	4	45.8	13332-13355/HCoV-OC43
554	Fethoc-TCG-TA(5)C-CCC-GA(5)A-CCC-G(6)TT-TAA-A(5)AA-NH2	24	4	45.8	13332-13355/HCoV-OC43
555	Fethoc-TCG-TA(5)C-CC(12)C-GAA(5)-CCC-G(6)TT-TA(5)A-AA(5)A-NH2	24	6	45.8	13332-13355/HCoV-OC43
556	Fethoc-Fethoc-ACC(Ph2)-CCG-A(5)AC-CCG(6)-TTT-AAA(5)-AA-NH2	20	4	45.0	13332-13351/HCoV-OC43
557	Fethoc-Fethoc-ACC(Ph2)-CCG-A(5)AC-C(Ph2)CG-TTT-AAA(5)-AA-NH2	20	4	45.0	13332-13351/HCoV-OC43
558	Fethoc-GCC(Ph2)-CAC-A(5)AG-CAT-A(5)GA-TTA(5)-CA-NH2	20	4	45.0	91-110/HCoV-OC43
559	Fethoc-AGA-TGC(12)-AA(5)T-TTA(5)-GG(6)T-GG(6)T-GC-NH2	20	5	45.0	19316-19335/HCoV-OC43
560	Fethoc-TGC(12)-AA(5)T-TTA(5)-GGT-GG(6)T-GC-NH2	17	4	47.1	19468-19484/SARS-CoV-2, HCoV-OC43
561	Fethoc-AGA-TGC(12)-AA(5)T-TTA(5)-GGT-GG(6)T-GC-NH2	20	4	45.0	19316-19335/HCoV-OC43
562	Fethoc-TTA(5)-AAC-G(6)GG-TTC-G(6)GG-G(6)T-NH2	17	4	52.9	13335-13351/HCoV-OC43
563	Fethoc-TTT-TTA(5)-AAC(12)-GGG(6)-TTC-G(6)GG-G(6)T-NH2	20	5	45.0	13332-13351/HCoV-OC43
564	Fethoc-TTT-TTA(5)-AAC-G(6)GG-TTC(12)-GGG-G(6)T-NH2	20	4	45.0	13332-13351/HCoV-OC43
565	Fethoc-C(12)TA-ACA-A(5)GA-GAT-CA(5)G-TGA-A(5)G-NH2	20	4	51.3	31-50/HCoV-OC43
566	Fethoc-CTA(5)-ACA-A(5)GA-GAT-CA(5)G-TGA-AG(6)-NH2	20	4	51.3	31-50/HCoV-OC43
567	Fethoc-GTT-TA(5)G-ATT-A(5)CA-AA(5)A-AGA-TC(12)-NH2	20	4	25.0	50-69/HCoV-OC43
568	Fethoc-G(6)TT-TAG-A(5)TT-ACA-AA(5)A-AGA(5)-TC-NH2	20	4	25.0	50-69/HCoV-OC43
FAM-PNA	FAM-(hexyl)2-AAG-TGT-ACC-TAA-AC-NH2	14	0	35.7	
FAM-OPNA	FAM-(Hexyl)2-AA(5)G-TG(5)T-A(5)CC(12)-TA(5)A-A(5)C-NH2	14	6	35.7	
081	Fethoc-A(5)GC-TCT-A(5)GA-CTT-A(5)CT-TTT-A(5)A-NH2	20	4	30.0	14527-14546/SARS-CoV-2
125	Fethoc-TTG-G(6)TT-TG(6)T-TAC-CTG(6)-GGA-A(5)G-NH2	20	4	45.0	16-35/SARS-CoV-2
129	Fethoc-A(5)CC-GCA-A(5)AC-CCG(6)-TTT-AAA(5)-AA-NH2	20	4	40.0	13460-13479/SARS-CoV-2

Supplementary Table2. Exact mass and purity of OPNA targeted HCoV-OC43 sequences.

Name	Exact Mass, m/z theor.[a]	Exact Mass, m/z obs.[b]	UPLC Purity(%)
1	6207.74	6207.78	98.55
2	6252.85	6253.22	99.65
3	6181.74	6181.81	98.43
4	6340.79	6341.13	97.94
5	5287.38	5287.43	98.76
6	5317.35	5317.35	99.19
7	4518.08	4518.11	97.51
8	4239.92	4239.94	97.87
9	4248.95	4248.98	99.02
10	4551.12	4551.15	99.29
11	5419.37	5419.41	98.62
501	6317.75	6317.90	94.95
502	6299.73	6299.81	98.45
503	6343.82	6343.87	94.45
504	6184.76	6184.83	97.73
505	5384.36	5384.50	95.53
506	4464.00	4464.12	97.49
507	4195.84	4195.88	95.46
508	4190.91	4190.97	96.89
509	4533.10	4533.15	94.33
510	4923.91	4923.96	94.08
511	5322.32	5322.39	98.94
512	5421.42	5421.49	90.80
513	5420.43	5420.47	96.82
560	5320.27	5320.27	94.71
514	6360.81	6360.86	97.59
559	6260.71	6260.69	93.28
561	6161.60	6161.64	94.11
515	6351.80	6351.85	98.19
516	4611.06	4611.17	98.15
517	4611.06	4611.10	93.74
518	5024.01	5024.06	93.17
519	5024.01	5024.07	97.34
520	5124.11	5124.16	95.23
521	4729.84	4729.74	93.35
522	4829.94	4829.97	97.62
523	4829.94	4829.93	96.26
524	4930.04	4930.07	96.82
525	5225.30	5225.25	96.41
526	5183.29	5183.33	96.84
545	6887.02	6886.98	93.48
546	7513.34	7513.29	93.37
547	8054.55	8054.52	94.43
548	7858.39	7858.32	95.08
527	4991.12	4991.14	98.01
528	5091.22	5091.24	98.77
538	5991.65	5991.73	97.81
539	6091.75	6091.83	97.66
540	6188.81	6188.91	97.65
554	7066.07	7066.02	94.75
555	7263.22	7263.14	93.10
537	6144.64	6144.67	98.42
553	7237.07	7237.03	91.76
562	5346.33	5346.34	95.94
563	6241.69	6241.62	94.55
564	6142.58	6142.57	93.48
529	6007.65	6007.47	99.23
530	6144.66	6144.72	94.85
549	7228.08	7228.05	97.13
550	7328.18	7328.15	97.17
531	6136.68	6136.69	97.34
551	7344.21	7344.15	93.40
565	6133.63	6133.63	93.94
566	6135.68	6135.67	93.32
532	6079.68	6079.56	98.05
533	6101.66	6101.54	97.88
552	7269.19	7269.16	94.92
567	6098.62	6098.60	94.71
568	6100.67	6100.66	94.37
534	6103.63	6103.69	98.77
535	6171.63	6171.74	99.63
536	6031.66	6031.37	98.86

Supplementary Figure 1. Alignment of the OPNA 11,HCoV-OC43, SARS-CoV and SARS-CoV-2 variants.

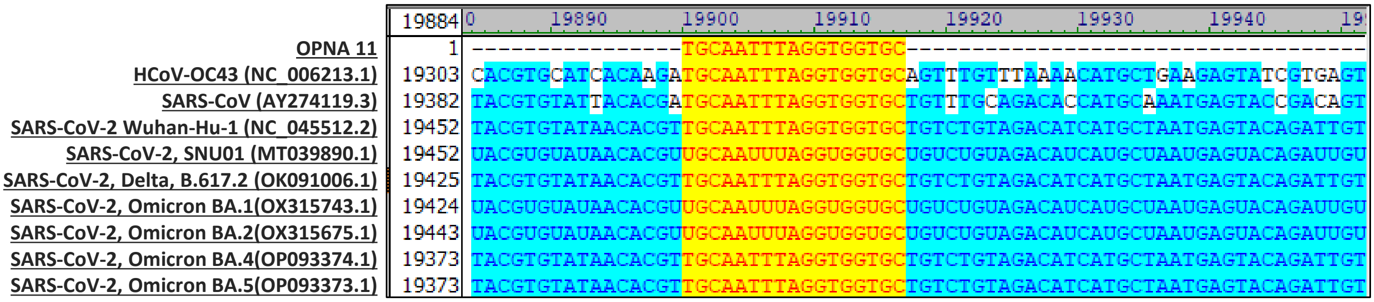

Supplement: Supplementary file 1 [file Data_Sheet_1.pdf]
